# Who will win where and why? An ecophysiological dissection of the competition between a tropical pasture grass and the invasive weed Bracken over an elevation range of 1000 m in the tropical Andes

**DOI:** 10.1371/journal.pone.0202255

**Published:** 2018-08-13

**Authors:** Johannes Knuesting, Marie Clara Brinkmann, Brenner Silva, Michael Schorsch, Jörg Bendix, Erwin Beck, Renate Scheibe

**Affiliations:** 1 Department of Plant Physiology, Faculty of Biology and Chemistry, Osnabrueck University, Osnabrueck, Germany; 2 Laboratory for Climatology and Remote Sensing, Faculty of Geography, Philipps-University of Marburg, Marburg, Germany; 3 Department of Plant Physiology, Faculty of Biology, Chemistry, and Geosciences, BAYCEER, University of Bayreuth, Bayreuth, Germany; El Colegio de la Frontera Sur, MEXICO

## Abstract

In tropical agriculture, the vigorously growing Bracken fern causes severe problems by invading pastures and out-competing the common pasture grasses. Due to infestation by that weed, pastures are abandoned after a few years, and as a fatal consequence, the biodiversity-rich tropical forest is progressively cleared for new grazing areas. Here we present a broad physiological comparison of the two plant species that are the main competitors on the pastures in the tropical Ecuadorian Andes, the planted forage grass *Setaria sphacelata* and the weed Bracken (*Pteridium arachnoideum*). With increasing elevation, the competitive power of Bracken increases as shown by satellite data of the study region. Using data obtained from field measurements, the annual biomass production of both plant species, as a measure of their competitive strength, was modeled over an elevational gradient from 1800 to 2800 m. The model shows that with increasing elevation, biomass production of the two species shifts in favor of Bracken which, above 1800 m, is capable of outgrowing the grass. In greenhouse experiments, the effects on plant growth of the presumed key variables of the elevational gradient, temperature and UV radiation, were separately analyzed. Low temperature, as well as UV irradiation, inhibited carbon uptake of the C4-grass more than that of the C3-plant Bracken. The less temperature-sensitive photosynthesis of Bracken and its effective protection from UV radiation contribute to the success of the weed on the highland pastures. In field samples of Bracken but not of Setaria, the content of flavonoids as UV-scavengers increased with the elevation. Combining modeling with measurements in greenhouse and field allowed to explain the invasive growth of a common weed in upland pastures. The performance of Setaria decreases with elevation due to suboptimal photosynthesis at lower temperatures and the inability to adapt its cellular UV screen.

## Introduction

**Fig 1:**
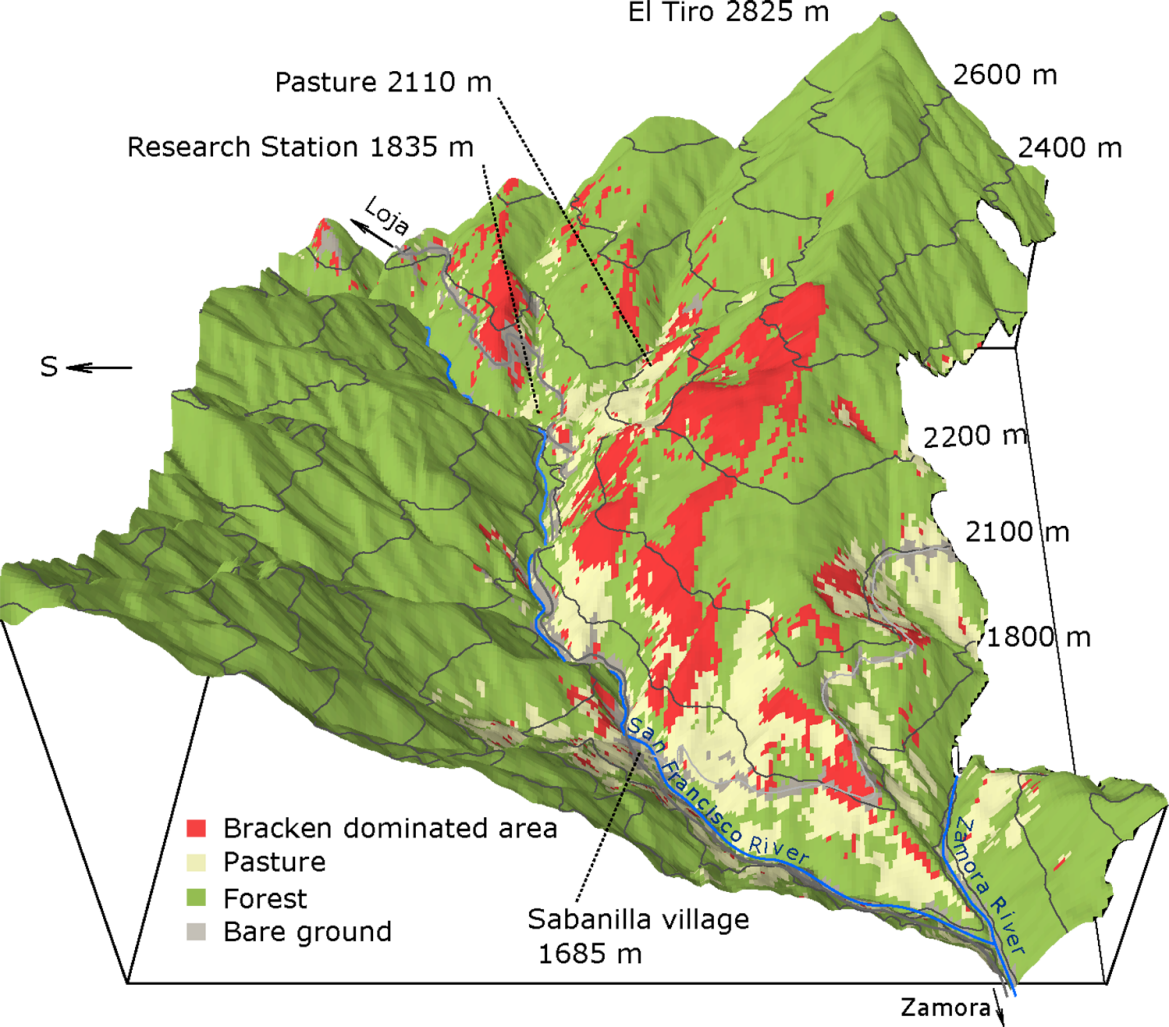
Invasion of Bracken into pastures as dependent upon elevation. A digital elevation model derived by airborne laser scanning is overlaid by the land-cover classification based on multispectral satellite data generated by Quickbird in November 2010 of the research area in the San Francisco valley in South Ecuador (from 1000 m to 2800 m elevation). The occurrence of Bracken, as detected by multispectral remote sensing, is shown in red, while pastures are in cream color. Study locations are labeled accordingly, and the elevation is given in m a.s.l.. The dominant grass on the pastures is Setaria.

In order to obtain grazing land in the humid tropical Andes of Ecuador, natural forest has widely been cleared by “slash and burn” up to an elevation of 3000 m. Since native forage grasses are absent in the understory of the dense mountain forest, introduced grass species, mostly from tropical Africa, are commonly used for pasture farming. At mid-elevations, *Setaria sphacelata* (referred to as “Setaria”), a C4-photosynthesis plant, is the dominating pasture grass which is transplanted manually as its seeds do not germinate under the local environmental conditions. It forms tussocks which propagate readily with tillers, and after 1 to 2 years, generates a more or less homogeneous pasture. Southern Bracken fern (*Pteridium arachnoideum* and *P*. *caudatum*), although indigenous to tropical South America [1], rarely occurs in the undisturbed forest, but rapidly invades areas on which fire has been used to clear the natural vegetation. It propagates by spores which are distributed by the wind. Additionally, it spreads via rhizomes which can reach down into the soil to a depth of 80 cm, but usually accumulate in the upper 20 cm of the soil forming a dense network from which single fronds develop. Bracken infests the planted pastures, running its rhizomes between the bulky rootstock of Setaria. In this communication, we worked with the diploid species *P*. *arachnoideum* (referred to as “Bracken”) which dominates in the uplands [1], whereas its allotetraploid relative *P*. *caudatum* is known as a lowland species [2]. Although Setaria and Bracken can coexist in a labile steady state on the pastures for some years, the common procedure of pasture rejuvenation by burning favors Bracken more than Setaria on the long run [3, 4]. As shown in Fig 1, Bracken is unevenly distributed on the slopes, and its abundance increases dramatically with elevation, while that of Setaria decreases [5, 6]. The relative competitive strength of two plant species is a multi-factorial syndrome of ecophysiological traits giving rise to similar or differing responses to the environmental settings. In an unsteady coexistence of species, i.e. a delicate balance between their biomass production, even small changes in environmental factors can cause a shift in favor of one of the competitors. Factors that are known to change with altitude in the Ecuadorian Andes are the intensity of irradiation, especially UV, amounts of precipitation, and temperature [7, 8]. Regarding the higher temperature optima of C4-plants (Setaria) relative to C3-plants (Bracken) [9], temperature, in particular, may be an ecologically important factor that can shift the competitive equilibrium between the species. Another ecophysiological trait that could affect the balance between both species in these pasture communities is the capability to accumulate protective, especially UV-absorbing compounds which shield the photosynthetic apparatus from excessive radiation [10]. In addition to their direct role in UV-protection, flavonoids are effective antioxidants and ROS scavengers, thus protecting the plant tissue also from secondary effects of UV radiation [11]. Taken together, flavonoids are essential for survival and success during colonization of new territory [12]. In various studies, quantitative responses of phenolic and flavonoid contents have been correlated with the elevation and the intensity of UV-irradiation [13–16].

For Bracken, a relatively high proportion of phenolic substances has already been identified [17, 18]. Alonso-Amelot et al. [18] showed a positive correlation between the content of these substances in the fronds and the elevation of its occurrence. No similar adaptations are known for Setaria. Being aware that other environmental factors such as soil quality, the amount of precipitation or allelopathic effects [19, 20] may also affect the steady state between Bracken and Setaria, we hypothesized that the decrease of temperature and the increase of UV radiation promotes the competitive growth of Bracken at higher elevations. To examine this hypothesis, we followed a four-step approach. First, we measured photosynthetic gas exchange in the field at different elevations. In a second step, we used the Southern Bracken Competition model (the SoBraCo model) [21], parameterized with the field data to determine annual photosynthetic carbon net uptake of both plants along an altitudinal gradient. Subsequently, the results of the model were compared with measurements under controlled close-to-nature-conditions in greenhouses. Finally, we investigated the UV screen in plant material collected in situ and compared it to that of plants grown under greenhouse conditions with supplemental UV irradiation. Here, the potential incidence of photoinhibition and the accumulation of phenolics (flavonoids and condensed tannins) protecting the plants from UV irradiation were of particular interest. Our study explains why using Setaria and presumably other C4-photosynthesis-type grasses as forage plants in tropical pastures above 1800 m a.s.l. elevation will not allow sustainable pasture farming without further measures of Bracken control [22, 23].

## Material and methods

### The plants: Setaria and Bracken

Setaria, also known as golden millet (*Setaria sphacelata* (Schumach.) Stapf & C.E. Hubb. var. *anceps* (Stapf) Veldkamp, Panicoideae, South African pigeon grass, Mequerón), native to tropical Africa, is today widespread in the tropics and subtropics in spite of the fact that it must be planted manually. In the Ecuadorian Andes, it is the common pasture grass at mid elevations. Bracken (Llashipa), “Southern Bracken” [24], is a neotropical complex consisting of the diploid *P*. *arachnoideum* (Kaulf.) Maxon and the allotetraploid *P*. *caudatum* (L.) Maxon [25] with one of its progenitors being *P*. *arachnoideum*, while the other is not quite clear [1]. Both species occur with a different distribution in the tropical Andes [26]: *P*. *arachnoideum* is considered an upland variant (1300–3200 m a.s.l.) [2]. In the research area, *P*. *arachnoideum* is the dominant species and has been used throughout in this study.

### The research area

The research area is located in the eastern range (Cordillera Oriental) of the South Ecuadorian Andes. The core area (Fig 1) is the 18 km long, deeply incised valley of the Rio San Francisco (3° 58’ S, 79° 4’ W) rising at the pass El Tiro (2825 m a.s.l.) in the West and merging with the Rio Zamora at the village Sabanilla (1685 m a.s.l.) in the East. The Research Station (Estación Cientifica San Francisco, ECSF) is located at 1835 m a.s.l. in the mountain rain forest (Fig 1). The research area comprises the natural tropical mountain rainforest on the southern slopes which is protected as part of the Podocarpus National Park, and a system of pastures, abandoned pastures, patches with exotic afforestation (Pinus, Eucalyptus), and remnants of the natural forest in a few gorges on the slopes. The pasture side of the valley is in the focus of this study. The research area had been rented from a local farmer by the Ecuadorian foundation Naturaleza y Cultura International (Loja, Ecuador, and San Diego, USA). The research was permitted by the Ministerio del Ambiente de Provincia Zamora-Chinchipe (Permit No. 007-IC-FAU/FLO-DPZCH-MA and 019-IC-FAU/FLO-DPZCH-MA).

The pastures suffer from a non-sustainable management, and most of them are abandoned after a few years [4]. The soils are cambisols which are commonly poor in phosphorus and nitrogen. On the pasture sites, the originally thick litter and the organic layer [27] have been removed upon clearing the forest by fire, and the nutrient input from the ash does not last long. The pH is slightly acidic (4.5 in water), and base saturation is low (between 11 and 17%) [28].

The climate of the area is tropical wet with a cloud frequency of 70% of the year with seven months of relative air humidity above 85% [7]. Table 1 lists climate variables for three automatic weather stations (AWS) in the area. The ECSF and the pasture AWS are on opposite slopes of the Rio San Francisco valley at different altitudes (1835 and 2110 m a.s.l., respectively), while the El Tiro station characterizes the climate of the pass altitude of the eastern Cordillera crest (2825 m a.s.l.). Temperature shows a clear decrease with altitude which is not the case for other variables in Table 1. This is because AWS point observations can differ from a simple elevation gradient due to its topographic position and land cover type. The lower average humidity on the pasture, for instance, is the result of a more extreme thermal and evaporation climate compared to that recorded by an AWS in the tropical mountain rain forest (ECSF) [29]. Moreover, it could be shown with spatial explicit data sets from satellites, weather radar and modelling that particularly the exposition towards the main airstream (windward, lee side) locally modifies cloudiness and thus irradiance [30], rainfall [31] and wind stress [32]. Nevertheless, Table 1 shows an increase of radiation extremes between 1835 and 2825 m a.s.l.. This is due to the outstanding irradiance levels during partly cloudy situations, particularly enhanced at higher elevations [33] which consequently goes along with higher UV stress in comparison to lower sites. While the radiation at higher altitudes is most of the time lower than at lower altitudes due to higher cloudiness, the short-term irradiance level at mountain crest areas is much higher during phases of scattered clouds and clear sky conditions resulting in a longer-term average radiation similar to that of lower areas (Table 1).

**Table 1:** Climate data from the three investigation sites as averages of 16 years (1998–2014).

Variable	ECSF (1835 m)	Pasture (2110 m)	El Tiro (2825 m)
Temperature (°C)	16.3	14.8	10.0
Rel. humidity (%)	83.7	80.8	93.9
Precipitation (mm/year)	1988	1573	1873
Max. precipitation (mm/year)	2196	1585	2462
Radiation (MJ/year)	4450	4220	4400
Max. radiation (MJ/year)	5435	4954	5845

### Sampling of field-grown plants

Almost all Setaria pastures are infested by Bracken; therefore, it was possible to collect plant material from both species from the same sites at 1300, 1800, and 2500 m a.s.l. in three cases. The uppermost samples (2600 m a.s.l.) were taken, however, not from a pasture, but from the roadside, since the former pastures have been abandoned for decades and were meanwhile overgrown by shrub vegetation. In each site, three leaves from different, healthy plants without obvious damage were collected. The geographical coordinates of the plots are given in S1 Table. Leaves of five different plants for each site and species were collected in the afternoon, cooled overnight in the refrigerator, transported in a cooling box and stored at -80°C. The stability of phenolic components of interest has been shown to be sufficiently high for these transport conditions [34].

### Growth conditions for greenhouse experiments

Plants grown from rhizomes (Bracken) or tillers (Setaria) taken at the pasture site in Ecuador were cultivated in pots with a diameter of 80 cm in soil containing 36% pumice, 36% compost, 18% peat, 9% sand and 1.5 g/l PolyCrescal. The greenhouse conditions were kept constant at PAR of 500 μmol m^-2^ s^-1^, at the given day temperatures with a 5°C decline of night temperature; rel. humidity was 55%. Plants (10 individuals of each of the experimental plants) were placed in parallel under UV and control conditions.) All of them were illuminated (12 h day length) with sodium vapor lamps (SON-T Agro 400 W, Philips, Eindhoven, The Netherlands). For the UV treatment, additional UV at 20°C was provided by ten UV-luminescent tubes (8F 100W, Philips) producing a UV intensity of 10 W m^-2^ as determined with the Smart Q Ultraviolet Sensor (Data Harvest Group Ltd., U.K.). The UV-B percentage in the monitored UV range was estimated to be 2% resulting in a radiation of 2.5 kJ m^-2^ per day. During the day, the UV lamps were switched on and off at intervals of 30 min. This setup was maintained for 70 days to simulate ecologically relevant conditions with varying cloud cover. For all experiments, at least three biological replicates of the respective plants and conditions were selected for analysis.

### Species-specific parameterization and set up of the photosynthesis model

In order to identify abiotic factors limiting the photosynthetic performance of Bracken and Setaria, gas-exchange rates were recorded in situ to obtain the responses of CO_2_ net uptake to varying light intensities and CO_2_ concentrations at 1835 and 2110 m a.s.l.. A portable photosynthesis-measurement system (LI-6400XT; LiCor Inc., Lincoln, U.S.A.) was used to estimate photosynthetic parameters (Table 2). Measurements were performed on intact leaves still connected with the plant, and the gas-exchange chamber was fixed on a tripod, oriented to maintain the natural orientation of the leaves. The samples in the field were randomly chosen. The parameters were averages of at least three measurements from different individuals in the field. All measurements were conducted from 11:00 a.m. to 5:00 p.m. local time, at external temperatures between 15–30°C. For the measurements, the temperature was set to 25°C and relative humidity to 55%. For the case that the rel. humidity was lower than 50% or higher than 65%, no data were recorded. Previous work of Long and Bernacchi [35] and Collatz et al. [36] were considered for the determination of photosynthetic parameters specific for C3- and C4-plants. The parameter V_Cmax_ represents the RubisCO activity and is derived from the initial slope of CO_2_-response curves of C3-plants. For C4-plants, the maximum carboxylation rate (V_max_) is derived from the saturated section of the A/C_i_ curves. Mitochondrial respiration (hereafter referred to as respiration) and the quantum yield are derived from light-response curves of CO_2_ net assimilation. Respiration is described as the point of interception with the Y-axis of a linear regression of light intensities below 200 μmol m^-2^ s^-1^. The slope of this linear regression equates quantum yield. The parameters with intrinsic elevational effects were further used to parameterize the SoBraCo model. The SoBraCo model [21, 28] is a canopy-photosynthesis model based on the two-big-leaf and the two-stream approaches [37].

**Table 2:** Parameters derived from field measurements of sunlit and shaded leaves of Setaria and Bracken at two altitudes.

Parameter	Site	Bracken		Setaria		Unity
		Light	Shade	Light	Shade	
V_max_	ECSF (1835 m)	-	-	20.72±2.80	29.16±0.01	μmol CO_2_ m^-2^ s^-1^
	Pasture (2110 m)	-	-	19.59±1.83	23.76±1.20	μmol CO_2_ m^-2^ s^-1^
V_Cmax_	ECSF (1835 m)	78.71±0.64	65.95±4.54	-	-	μmol CO_2_ m^-2^ s^-1^
	Pasture (2110 m)	62.98±5.52	62.37±12.85	-	-	μmol CO_2_ m^-2^ s^-1^
Respiration	ECSF (1835 m)	1.41±0.19	0.57±0.11	1.71±0.15	2.30±0.12	μmol CO_2_ m^-2^ s^-1^
	Pasture (2110 m)	1.36±0.13	0.64±0.25	1.98±0.36	1.55±0.20	μmol CO_2_ m^-2^ s^-1^
Quantum yield	ECSF (1835 m)	0.042±0.004	0.040±0.001	0.052±0.003	0.062±0.001	mol CO_2_ mol^-1^ quanta
	Pasture (2110 m)	0.039±0.004	0.039±0.002	0.038±0.002	0.061±0.006	mol CO_2_ mol^-1^ quanta

V_max_, maximum carboxylation rate of C4-plants; V_Cmax,_ max. RubisCO activity of C3-plants; Respiration, mitochondrial respiration. ECSF, research station. It should be noted that the derived parameters intrinsically integrate the effects of local growth conditions like nutrient status of the plants. Each parameter is the mean ±SEM (n ≥3).

In addition to the species-specific photosynthetic parameters, the SoBraCo canopy-photosynthesis model uses forcing with realistic data gathered by automatic weather stations [21]. Forcing data include global incident radiation, air temperature and relative humidity, atmospheric pressure, wind speed, soil moisture and soil temperature. At 2110 m a.s.l., the following setup was used to record the corresponding parameters: pyranometer CNR1 (Kipp and Zonen, Delft, The Netherlands); thermometer and hygrometer MP100A (Rotronic, Ettlingen, Germany); barometer CS100, TDR reflectometer CS616, Thermocouple TCAV at 10 cm depth (all from Campbell Scientific, Logan, UT, U.S.A.) and anemometer (Vector Instruments, Rhyl, North Wales, U.K.). The setup of the weather stations at the other elevations was described in Rollenbeck et al. [38, 39], and the climate parameters were extrapolated for 1835 m a.s.l.. The photosynthesis parameters derived from field measurements at the pasture site were used for the simulation at the El Tiro site (2825 m a.s.l.). Forcing data used in the simulation are hourly averaged weather data (available between the years 1999 and 2014).

### Preparation of pigment extracts for TLC, colorimetry, and HPLC/MS

Leaf extracts for analyses by colorimetry, thin-layer chromatography, and mass spectrometry were prepared with aqueous HCl-MeOH (MeOH:conc. HCl:H_2_O (90:1:1) as extraction medium. For extraction, 0.1 g of frozen leaf tissue (-80°C) was transferred into a 2.0-ml graduated skirted tube with a screw cap (Starlab, Hamburg, Germany), and 1 ml extraction medium was added together with metal beads (Omni International, Inc., Tulsa, U.S.A.). The extraction was achieved with the Omni Bead Ruptor 24 (Omni International, Inc., Tulsa, U.S.A.) at 4.7 m/s for 3 x 3 min with pauses of 45 s in between. The extract was centrifuged at 10,000 *xg* for 10 min. This extract was used directly for TLC analyses (see below).

An aliquot of the supernatant was defatted by the addition of petroleum ether (one-third of the sample volume). This mixture was transferred to a separating funnel and shaken vigorously. The lower phase was collected and subjected to the same procedure two more times, where the upper phase was discarded each time so that the total volume of petroleum ether equaled the initial total sample volume. HPLC and subsequent mass spectrometry analyses (see below) were performed with 80 μl of the defatted extracts.

As an alternative of sample extraction used for HPLC/MS analysis, flavonoids were harvested from intact leaves using phosphate-coated nanoparticles that were prepared as described by Kurepa et al. [40]. In short, the TiO_2_ nanoparticles were synthesized as described in Abbas et al. [41] and then functionalized with phosphate. For nano-harvesting, plant parts (100 mg) were submerged in a suspension (0.1 g/ml suspension) of the phosphorylated ultra-small TiO_2_ nanoparticles (NP) and incubated for 2 h at room temperature in darkness. Particle size (20 nm diameter) and crystal form (anatase) of NP used for the experiments were confirmed in each freshly made preparation (M. Haase, Chemistry Department, Univ. Osnabrueck).

### Thin-layer chromatography of phenolic compounds

Extracts obtained with aqueous HCl-MeOH were used directly for thin-layer chromatography (TLC). Ten μl for Setaria or twenty μl for Bracken of each sample were separated on high performance (HP)TLC silica plates (Merck, Darmstadt, Germany), applying 10 μl of 1 mM solutions of rutin and apigenin as standards. The chromatograms were developed with n-butanol:acetic acid:H_2_O (4:1:5, upper phase, BAW). After drying, they were sprayed with Naturstoff reagent A (diphenyl boric acid-2-aminomethyl ester, DPBA) and inspected under UV light (302 nm). Soluble oligomeric and polymeric proanthocyanidins were separated in the same way using epicatechin (1 mM, 10 μl) as standard. Visualization of bands was achieved by spraying with 0.1% dimethylaminocinnamaldehyde (DMACA, from Sigma stock solution 1% in 0.1 N HCl) diluted in 3 N HCl/50% aqueous ethanol [42].

### Colorimetric assays

Total flavonoids in aqueous HCl-MeOH extracts were detected with AlCl_3_ as the transition metal using rutin as a standard [15]. Total phenolics were determined with the Folin-Ciocalteu assay using gallic acid as a standard [43]. Insoluble proanthocyanidins were dissolved by butanolysis and quantified using a calibration curve with epicatechin according to Pang et al. [44]. Specific staining of condensed tannins in thin sections and tissue powder was achieved with 1% DMACA solution in 0.1 N HCl.

### HPLC and mass spectrometry of phenolic compounds

Both, defatted extracts and nano-harvested material were loaded onto a trap column (Acclaim PepMap C18, 5 μm, 0.1 × 20 mm, Thermo Scientific, Sunnyvale, CA, U.S.A.) and washed. The trapping column was switched in line with a separation column (Acclaim PepMap C18 2 μm, 0.075 × 150 mm, Thermo Scientific). Subsequently, bound substances were eluted by gradually changing the mixture of solvent A (99% water, 1% acetonitrile, 0.1% formic acid) and solvent B (80% acetonitrile, 20% water, and 0.1% formic acid) from 100:0 to 20:80 within 45 min. The flow rate was kept constant at 0.3 μl/min. Successively eluted compounds were analyzed with i) an UV detector 330 nm, and ii) an ESI-ion trap (Amazon ETD Speed with a captive spray ionization unit, Bruker Corporation, Billerica, MA, U.S.A.) for measuring the masses of the intact molecules as well as the masses of the fragments, which were generated by collision-induced dissociation (CID) of the corresponding parent ion.

For the identification of compounds eluting in the UV (330 nm)-absorbing peaks, MassBank (www.massbank.jp) was used [45]. For final decisions, the expected masses after the second cycle were taken into account.

### Western blot analysis

Leaf tissue of the experimental plants was ground in liquid nitrogen, and 1 ml protein extraction buffer (0.1 M Tris-HCl, pH 7.6, 50 mM NaCl, 2% SDS) was added per 0.1 g together with a pint of insoluble polyvinylpolypyrrolidone (PVPP). The samples were kept in an ice-bath for 10 min while gently shaken. After centrifugation for 10 min at 16,000 *xg*, the supernatant was collected, and protein concentration was determined using the bicinchoninic acid assay (Pierce) with BSA as a standard. After separation by denaturing polyacrylamide-gel electrophoresis (SDS-PAGE), the gel was blotted onto a nitrocellulose membrane, and incubated overnight with the primary antiserum against the D2 subunit of PSII (Agrisera, Vännäs, Sweden) (diluted 1:10,000). After washing three times with TBS buffer, the membrane was incubated for 1 h with the anti-rabbit IgG linked to alkaline phosphatase at 1:3000 dilution (BioRad, Munich, Germany) and bands were visualized by adding 5-bromo-4-chloro-3-indolyl phosphate (BCIP) and nitro blue tetrazolium chloride (NBT) as substrate system for the phosphatase assay to produce the insoluble purple product on the membrane.

### Determination of photosynthetic efficiency after UV treatment

Chlorophyll a fluorescence was analyzed in three independent samples for each treatment to determine the photosynthetic efficiency using the pulse-amplitude-modulation fluorimeter FluorCam 800MF (Photon Systems Instruments, Czech Republic). Before measuring, leaves were kept in darkness for 10 min. The shutter was set to 10 ms, and the sensitivity to 13%. The obtained data were analyzed with the software FluorCam 7.

## Results

### Photosynthetic performance of Bracken and Setaria growing at the different elevations

**Fig 2:**
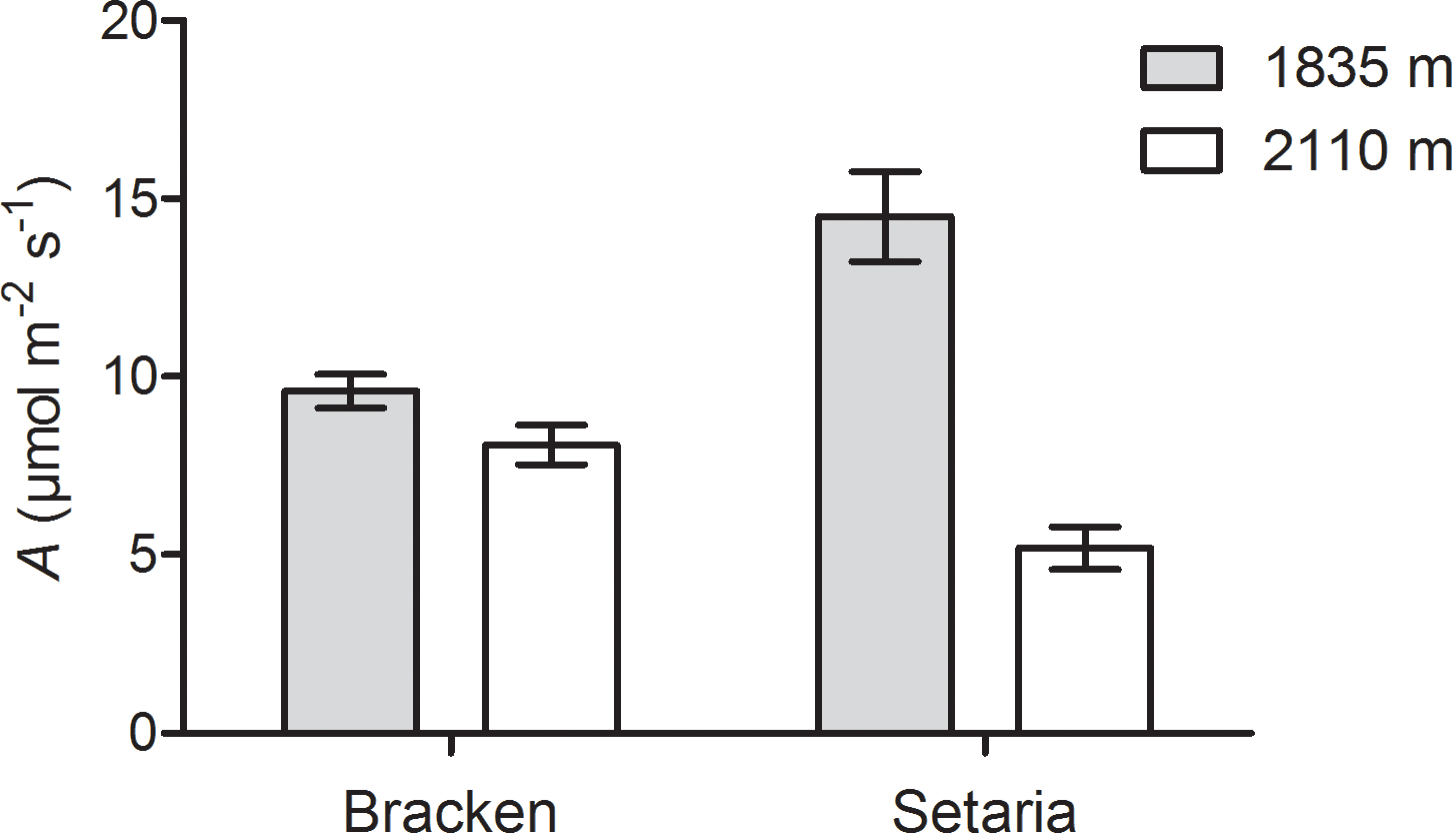
Photosynthetic efficiency of Bracken and Setaria under field conditions. CO_2_-assimilation rates (*A*) of both species were measured in the field at 1835 m a.s.l. (site 1, gray bars) and 2110 m a.s.l. (site 2, white bars). *A* was determined under physiologically relevant conditions of the study site at light intensities of 1200 μmol quanta m^-2^ s^-1^, internal CO_2_ concentrations of 200 μmol CO_2_ mol^-1^ and 25°C. The graph shows the mean ±SEM (n ≥3). According to the Student *t*-test, the differences between all *A* values are significant (*p* ≤ 0.01).

Photosynthetic carbon acquisition (*A*) responds to environmental variables such as light intensity, temperature, moisture, and internal CO_2_ concentration. To assess the photosynthetic capacity of both species in their natural environment, the response of CO_2_ net uptake to light intensity and CO_2_ concentration was determined at 1835 (site 1) and 2110 m a.s.l. (site 2) differing in elevation by 275 m and annual average temperature by 1.5°C [7, 46]. The sporadic local temperature challenges apparently result in major differences of the effects on the over-all performance of the plants despite of the determined annual average. Under natural field conditions, light-saturated photosynthetic CO_2_ net uptake of Setaria decreased dramatically (by almost two-thirds) between 1835 and 2110 m a.s.l. elevation, while that of Bracken was lower by only one-fifth (Fig 2). Thus, a significant decrease of *A* was apparent for Setaria at the upper site, whereas *A* of Bracken decreased only slightly when measured under comparable conditions, resulting in a value twice as high than in the C4-plant Setaria.

The observed decrease in photosynthetic efficiency of Setaria with increasing altitude was partly caused by its response to different internal CO_2_ concentration (C_i_) at saturating light intensity (S1 Fig). At the lower site 1 (1835 m a.s.l.), *A*_max_ of Setaria was achieved with 400 μmol external CO_2_ mol^-1^, whereas at the upper site, about 1200 μmol CO_2_ mol^-1^ were necessary to reach saturation.

In summary, the photosynthetic performance of the C3-plant Bracken decreased to a lesser extent with increasing elevation than that of the C4-plant Setaria. The advantage of C4-photosynthesis of Setaria over the C3-type of Bracken disappeared with altitude (at 2110 m a.s.l.) and was apparent only at the lower site (1835 m a.s.l.).

### Modelled photosynthetic performance of the C3-plant Bracken and the C4-plant Setaria along an elevational gradient

**Fig 3:**
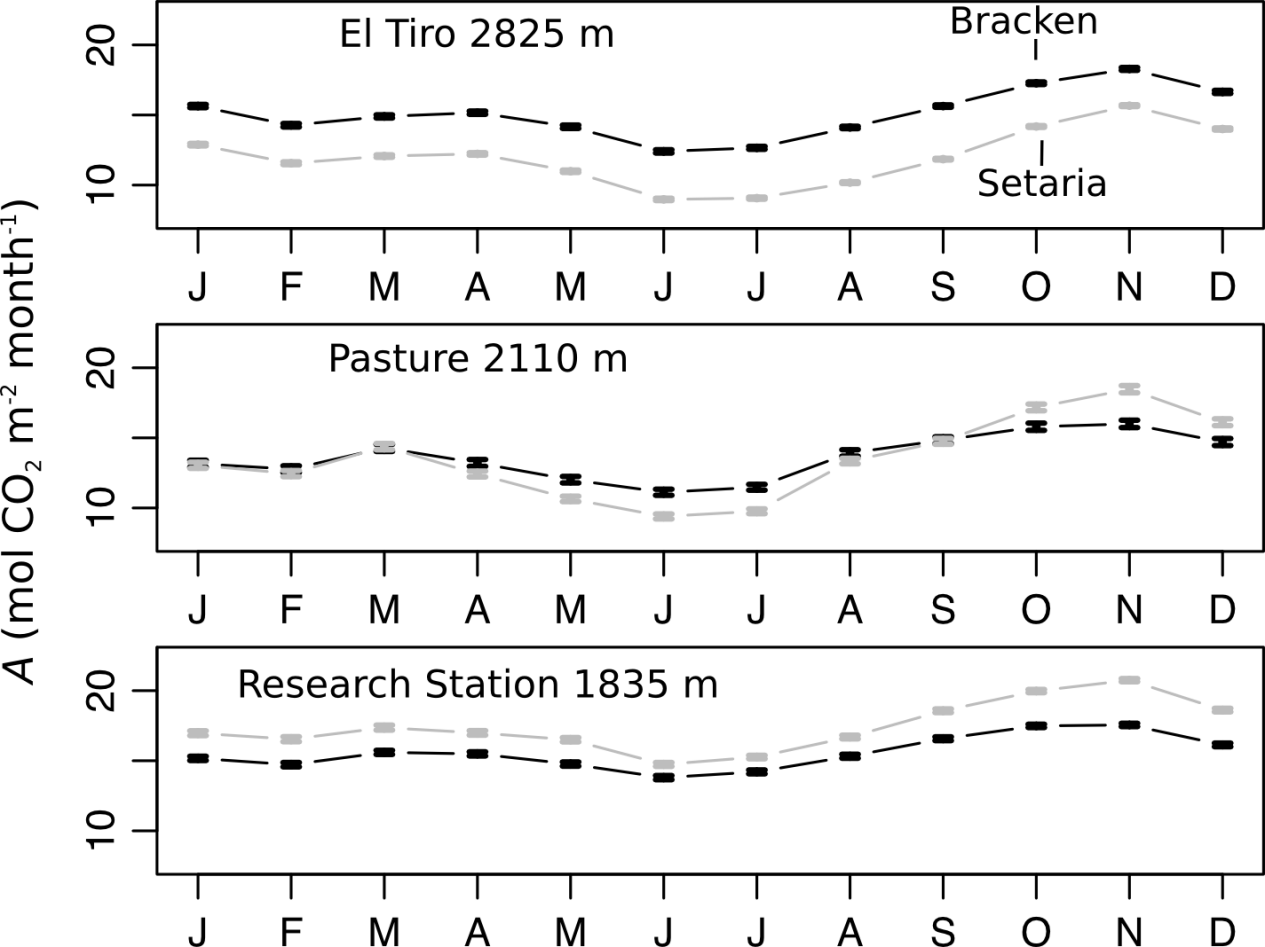
Simulation of canopy photosynthesis of Bracken and Setaria along an altitudinal gradient. *A* was simulated by using the SoBraCo model for Bracken (black lines) and Setaria (gray lines) at three different elevations. Species-specific parameters were derived from gas-exchange measurements in the field (Table 2). At the given elevations, weather stations recorded the climate data which were used for the site-specific forcing of the model. For the simulation, the climate data were averaged over the years 1999–2014 for each weather station, and the model was run in hourly time intervals. The error bars show the SEM of the monthly average CO_2_ assimilation for hundred model runs for each station and species.

In order to detect whether photosynthetic capacity is directly triggering the higher fitness of Bracken with the increasing elevations, the photosynthetic carbon acquisition was simulated at three different sites for the two species in high temporal resolution over an entire year. To that end, the SoBraCo model for photosynthetic carbon acquisition [21] was parameterized using the data of Table 2. The model was forced with the climate data averaged over 16 years in monthly resolution (incident radiation, UV, temperature, rel. humidity, wind speed, atmospheric pressure, soil moisture, and soil temperature) for the 3 sites at 1835, 2110, and 2825 m a.s.l. elevation, respectively (Fig 3).

At the highest site (2835 a.s.l., site 3), the model revealed that photosynthetic carbon gain of Bracken was consistently superior to that of Setaria during the entire year with calculated 182 (±1) mol CO_2_ m^-2^ and 144 (±1) mol CO_2_ m^2^ for the fern and the grass, respectively. A similar trend was found at 2110 m a.s.l. (site 2). Intriguingly, in this intermediate altitude, an exception was observed for the less rainy months. From September to December, photosynthesis of Setaria was higher than that of Bracken. This indicates that at mid elevations (site 2), Setaria as a C4-plant temporarily exceeded the performance of Bracken by 8±1% in the drier period. On the other hand, in the very wet period from April to July, carbon net uptake of Setaria was lower by 12±1% than that of Bracken. At 1835 m a.s.l. (site 1), Setaria was superior to Bracken except in the wet period from June to August when both plants photosynthesized at similar rates.

Taken together, for Setaria, the calculated annual net carbon gains per year at 1835, 2110, and 2825 m a.s.l. significantly decreased stepwise from 209 (±8) to 162 (±1), and to 144 (±1) mol CO_2_ m^-2^, respectively. At the same time, net carbon gain of Bracken did not show a significant reduction with 187 (±8), 165 (±1), and 182 (±1) mol CO_2_ m^-2^, respectively, at the 3 different elevations. In particular, the annual net carbon gain of Setaria and Bracken at the lowest (site 1) and the highest site (site 3) is significantly different. The SoBraCo model confirmed the assumption that the climatic conditions along an elevational transect affect the rate of photosynthesis of Bracken and Setaria differently, thus shifting the steady state of biomass production in favor of Bracken.

### Effects of temperature and UV on Setaria and Bracken

#### Influence of temperature on photosynthetic performance

**Fig 4:**
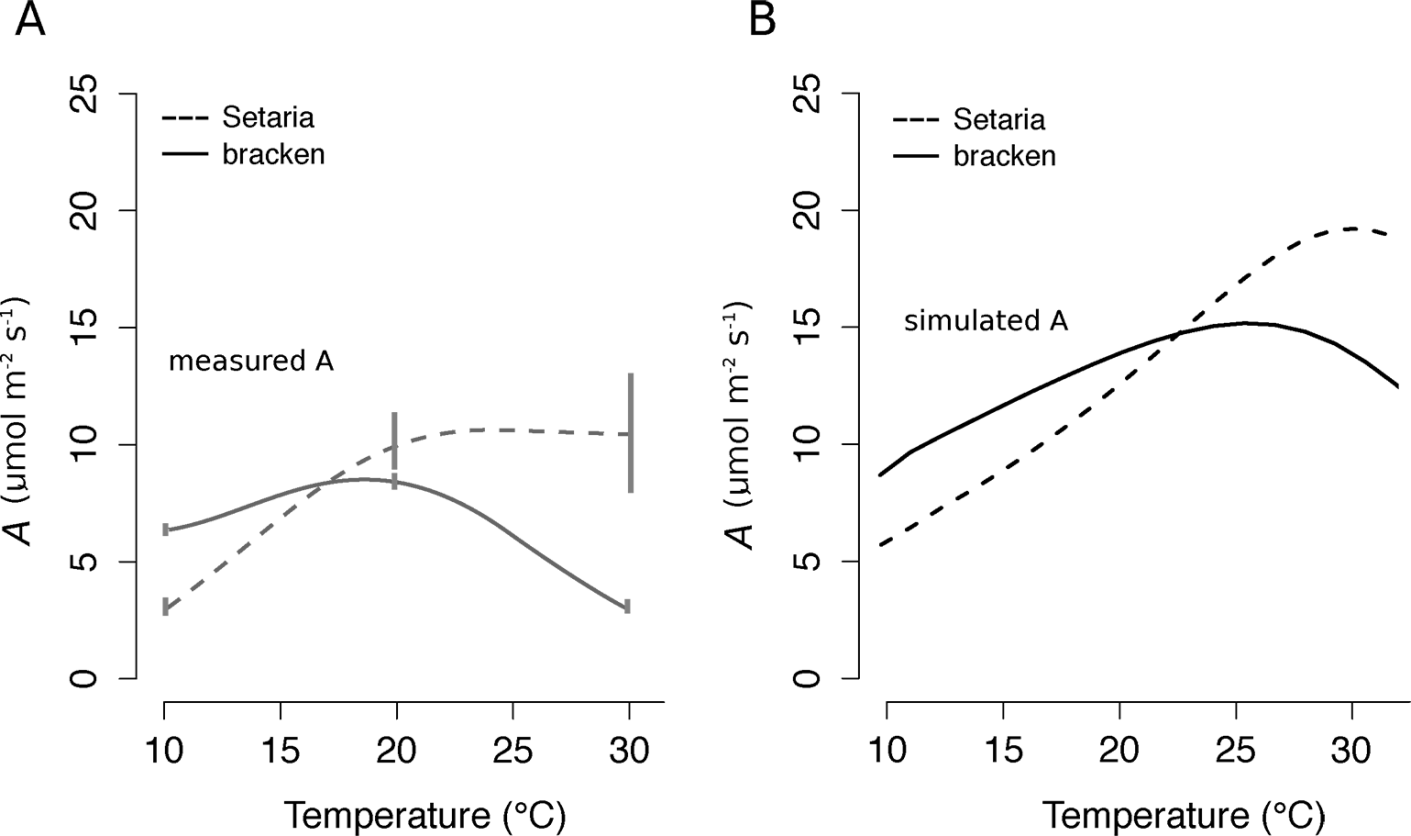
Temperature response of photosynthesis in Bracken and Setaria. **(A)** Bracken (solid lines) and Setaria (broken lines) plants were cultivated in greenhouses at 10, 20, and 30°C at 500 μmol quanta m^-2^ s^-1^. *A* of both species was measured at the growth temperatures at saturating light (1200 μmol quanta m^-2^ s^-1^), and 400 μmol CO_2_ mol^-1^ (gray lines; n ≥3 for each temperature; error bars show ± SEM). The measured CO_2_ uptake at the different temperatures was fitted by polynomial regression analysis. **(B)** The temperature response of photosynthesis for Setaria and Bracken was modeled including a temperature as a variable. Conditions were light saturation (1200 μmol quanta m^-2^ s^-1^), ambient CO_2_ (400 μmol CO_2_ mol^-1^), and the photosynthesis parameters from the field (Table 2).

In order to separate the influences of temperature and UV on the growth of Bracken and Setaria, both species were grown under controlled conditions in the greenhouse at 500 μmol quanta m^-2^ s^-1^ at 10, 20 and 30°C. The photosynthetic CO_2_ net uptake was determined under the saturating light intensity of 1200 μmol quanta m^-2^ s^-1^ at the same three temperatures. Setaria showed maximum rates of *A* above 22°C, whereas the temperature optimum of Bracken photosynthesis was between 17 and 20°C (Fig 4A).

In order to estimate a temperature optimum of *A* for the plants grown in the pasture, the model was forced with saturating light intensities (Fig 4B). In the case of Bracken, a broad temperature optimum of around 25°C and in the case of Setaria, a narrower one of 30°C was apparent. The model confirmed that temperatures below 22°C limit photosynthesis of Setaria to a greater extent than that of Bracken. High light intensities and low temperatures are typically occurring on the highland pastures, especially in the morning [47]. Therefore, the microclimate of pastures above 1800 m a.s.l. elevation promotes Bracken, because the average annual temperature (below 16°C) appears to be too low for Setaria to successfully compete with Bracken.

#### Influence of UV on growth and photosynthesis in the greenhouse

**Fig 5:**
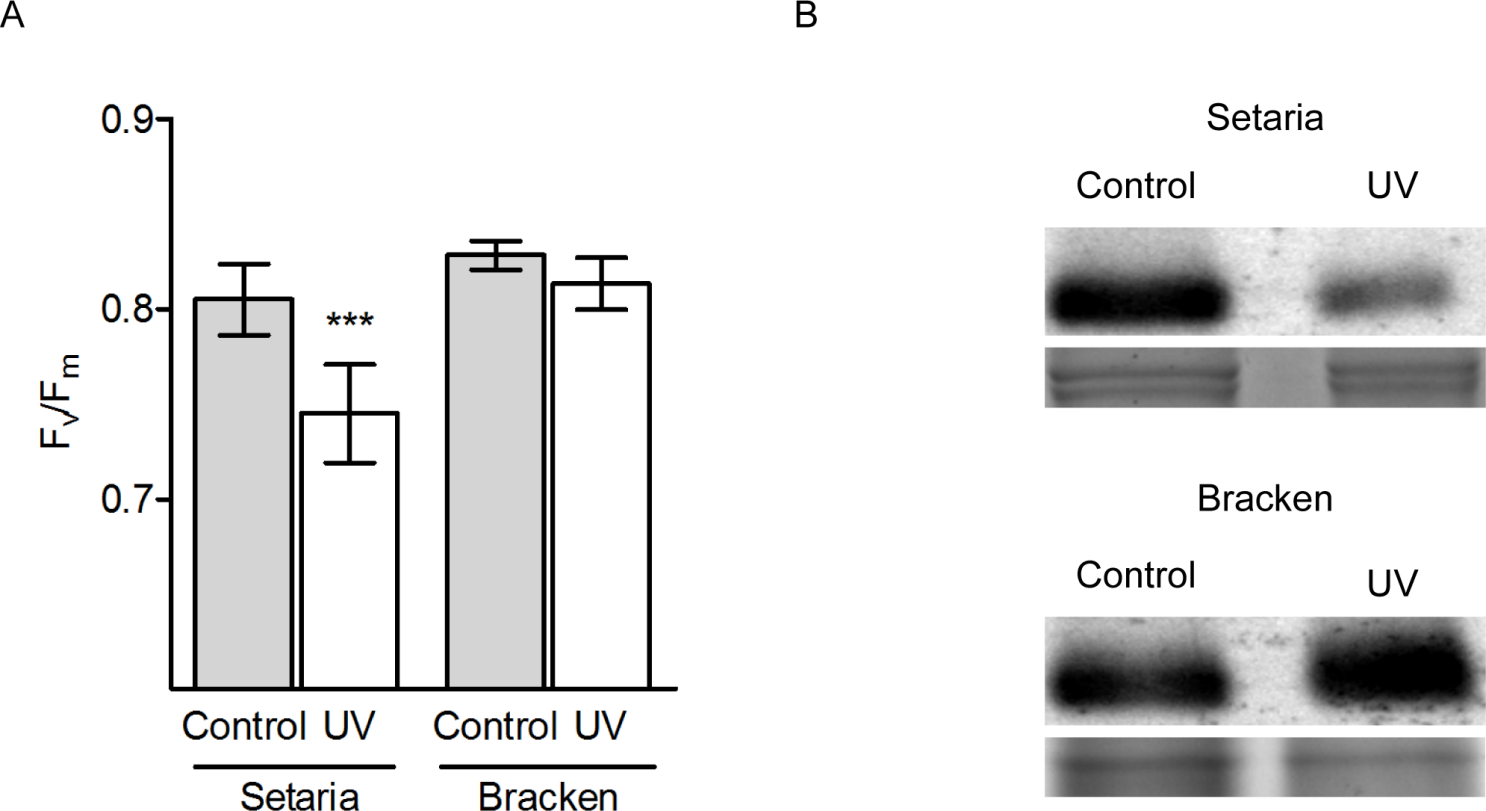
Influence of supplemental UV in the greenhouse on growth and photosynthesis of Setaria and Bracken. **(A)** F_v_/F_m_ was determined for both species after UV-light exposure using the FluorCam. The graph shows the mean ± SEM of three independent experiments. A statistical test was performed using the unpaired *t*-test, *** indicates *p* values ≤ 0.001). **(B)** Immunodetection of D2 protein after UV-light exposure and in controls. The Coomassie stain was used as protein loading control. Setaria and Bracken were exposed to UV light, with twelve 30-min periods of UV per day for 70 days (UV), and without additional UV-light exposures (Control). The plants were grown at 20°C.

In another set of experiments, the effect of increased UV radiation on photosynthesis was investigated under controlled conditions in the greenhouse. A severe growth limitation was observed for Setaria after 60 days of intermittent UV exposure (S2 Fig), while the growth of Bracken was not affected. No differences in the maximum photosynthetic quantum yields (F_v_/F_m_), were observed between the reference and UV-exposed Bracken leaves (control 0.82 vs. UV-treated 0.81) (Fig 5A) which were in the optimal range [48]. Supporting protein immunoblotting analysis after UV exposure resulted in an unchanged content of D2 protein of the PS II reaction center. Therefore, a photoinhibition effect after UV exposure can be excluded for Bracken. In contrast, Setaria showed a significant reduction of F_v_/F_m_ concomitant with lower D2 protein content in UV-treated plants (Control 0.81 vs. UV 0.74), suggesting UV-triggered degradation of PSII (Fig 5B). Overall, the UV-exposure experiments demonstrate a significant impact of the UV irradiation on the photosynthetic performance of Setaria, but not of Bracken.

### UV-screen composition in greenhouse- and field-grown plants

#### Soluble compounds

Cellular UV screens of Bracken and Setaria were examined after exposure to UV irradiation in the greenhouse and compared to those of control plants grown without additional UV light. A significant increase in the UV-A and UV-B range of the absorption spectra of aqueous HCl-methanol extracts from Bracken leaves of UV-treated, and untreated plants was apparent, which was not the case for extracts from Setaria (S3 Fig).

**Fig 6:**
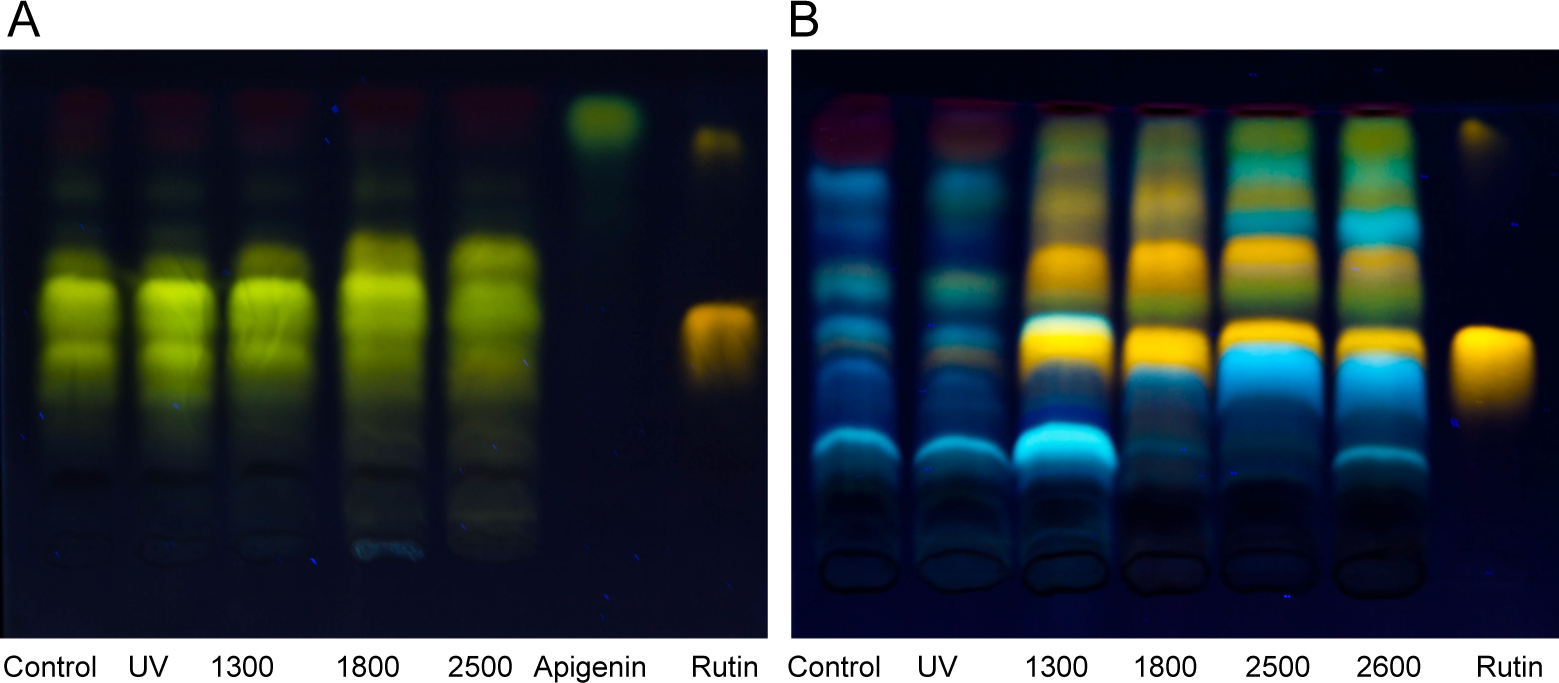
Comparison of the flavonoid pattern of Setaria and Bracken grown in greenhouses and the field. Thin-layer chromatography on an HPTLC-silica plate was performed with aqueous HCl-MeOH extracts of Setaria **(A)** and Bracken **(B)** leaves. The chromatograms were stained with Naturstoff reagent A and inspected under UV light. The origin of the samples is indicated in the figure (Control: greenhouse without additional UV radiation; UV: UV treatment in the greenhouse). The field samples are marked with the corresponding elevations in m a.s.l.. Apigenin and rutin were applied as markers in A; rutin was used in B. The fast moving compound is an impurity of the test substance rutin. The chromatograms are representative examples from n≤3.

To get more insight into specific differences, we analyzed the composition of flavonoids and other phenolic compounds of leaves from plants grown in the greenhouse and from the field. Thin-layer chromatography (TLC) of the leaf extracts and subsequent staining with Naturstoff reagent A revealed species-specific patterns of compounds for both species (Fig 6). In general, the pattern of bands obtained from samples of Setaria was less diverse and nearly independent of origin and treatment of the samples. In contrast, samples taken from Bracken showed highly diverse patterns on TLC. For further identification of secondary metabolites that are potentially involved in UV protection, we purified the major compounds using defatted aqueous HCl-MeOH extracts (E) as well as nanoparticle extractions (N) and subsequently analyzed these by HPLC and mass spectrometry (S2 and S3 Tables for Setaria and Bracken).

The most prominent flavonoid of Setaria leaves was identified as the flavanone glycoside poncirin (Fig 6A, S2 Table). Also, a grass-specific phenolic compound, namely the flavonolignan precursor tricin [49, 50], was present in all Setaria samples. Moreover, widespread flavones and their glycosides, such as luteolin/orientin, vitexin-rhamnoside, saponarin, and scoparin, were found in all Setaria samples, irrespective of the UV treatment. The only flavonoid in Setaria leaves responding to UV treatment and to growth at higher elevations, respectively, was the flavanone-rutinoside hesperidin. Furthermore, only in field-grown samples, a cyanidin glucoside was found that appears to be part of a larger molecule that also contains cinnamic acid. In summary, almost no effect of the UV treatment could be detected in the flavonoid patterns of the Setaria leaves.

In contrast, chromatograms of the extracts from all Bracken plants showed a large number of differently colored bands after staining with Naturstoff reagent A. The intensity of some of the bands increased in extracts of samples from higher elevations (Fig 6B). The field-grown samples contained higher amounts of soluble flavonoids than those from the greenhouse. Several blue fluorescing bands suggest the presence of hydroxycinnamic acid derivatives. The flavonoid patterns of the Bracken samples from the field and from the greenhouse differred to some extent. Especially quercetin and its glycoside rutin, which produce prominent orange bands in the field samples, appear to be of low abundance or even absent in the samples from the greenhouse. Mass spectrometric analysis, however, revealed the occurrence of quercetin and rutin exclusively in UV-exposed Bracken in the greenhouse, and in the field-grown samples (S3 Table). The flavanone glycosides hesperidin and poncirin appear to occur only in the field-grown samples. In contrast, kaempferol-7-O-neohesperidoside, and the luteolin glucoside orientin were present in all samples. An anthocyanidin derivative, namely cyanidin 3-O-[2''-O-(2‴-O-(sinapoyl) xylosyl) 6''-O-(p-O-(glucosyl) p-coumaroyl) glucoside] 5-O-glucoside was present in samples from higher elevations. The typical epicatechin compound proanthocyanidin B1 was exclusively found in Bracken from higher elevations, indicating the formation of polymeric structures of condensed tannins [51]. In contrast to Setaria which did not exhibit any pronounced reaction to the UV treatment or to the change of environmental conditions along the elevation gradient, the flavonoid patterns of Bracken apparently responded to these factors.

**Fig 7:**
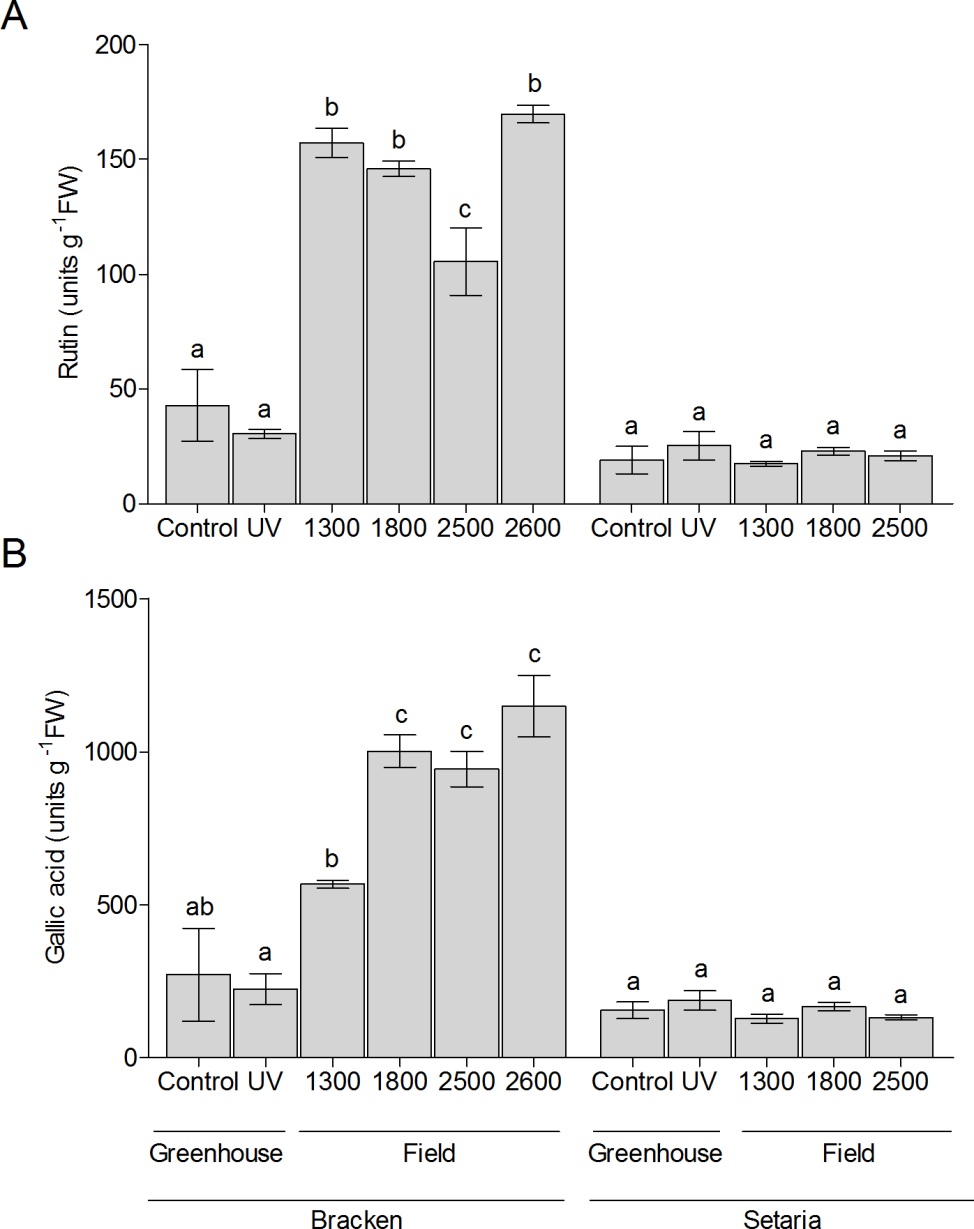
Total flavonoid and total phenolic contents in greenhouse- and field-grown Setaria and Bracken. **(A)** Flavonoid content was determined from field-grown and greenhouse-grown plants according to Kishore et al. [15] with rutin for calibration. **(B)** Total phenolics were determined according to Singleton and Rossi [43] with gallic acid for calibration. The graphs show the mean ± SEM of three biological replicates. The letters a, b and c indicate the level of significant differences (p ≤ 0.05) towards each other. Significance was calculated by ANOVA followed by Tukey’s pairwise comparisons.

For quantification of the flavonoid content, colorimetric determination of total flavonoids was performed (Fig 7A). Bracken samples from the field contained a three- to four-fold content of flavonoids as compared to those from the greenhouse, however without clear correlation with the elevation of the sites. Also, UV treatment in the greenhouse did not result in a substantial change of the flavonoid content as determined with the complexing reagent AlCl_3_. In Setaria, the levels of flavonoids were constantly low in all samples. To take into account not only members of the flavonoid group of aromatic compounds but all UV-absorbing compounds, total phenolics were quantified (Fig 7B). In Setaria, field-grown and greenhouse samples contained more or less similar, but rather low amounts of phenolic substances when estimated as gallic acid equivalents. In contrast, a tendency of increased phenolic content was detected in Bracken along the altitudinal gradient. The greenhouse samples, on the other hand, yielded lower values irrespective of the additional UV radiation. This result suggested an examination of insoluble, UV-screening compounds in the leaves.

#### Insoluble UV-screens

**Fig 8:**
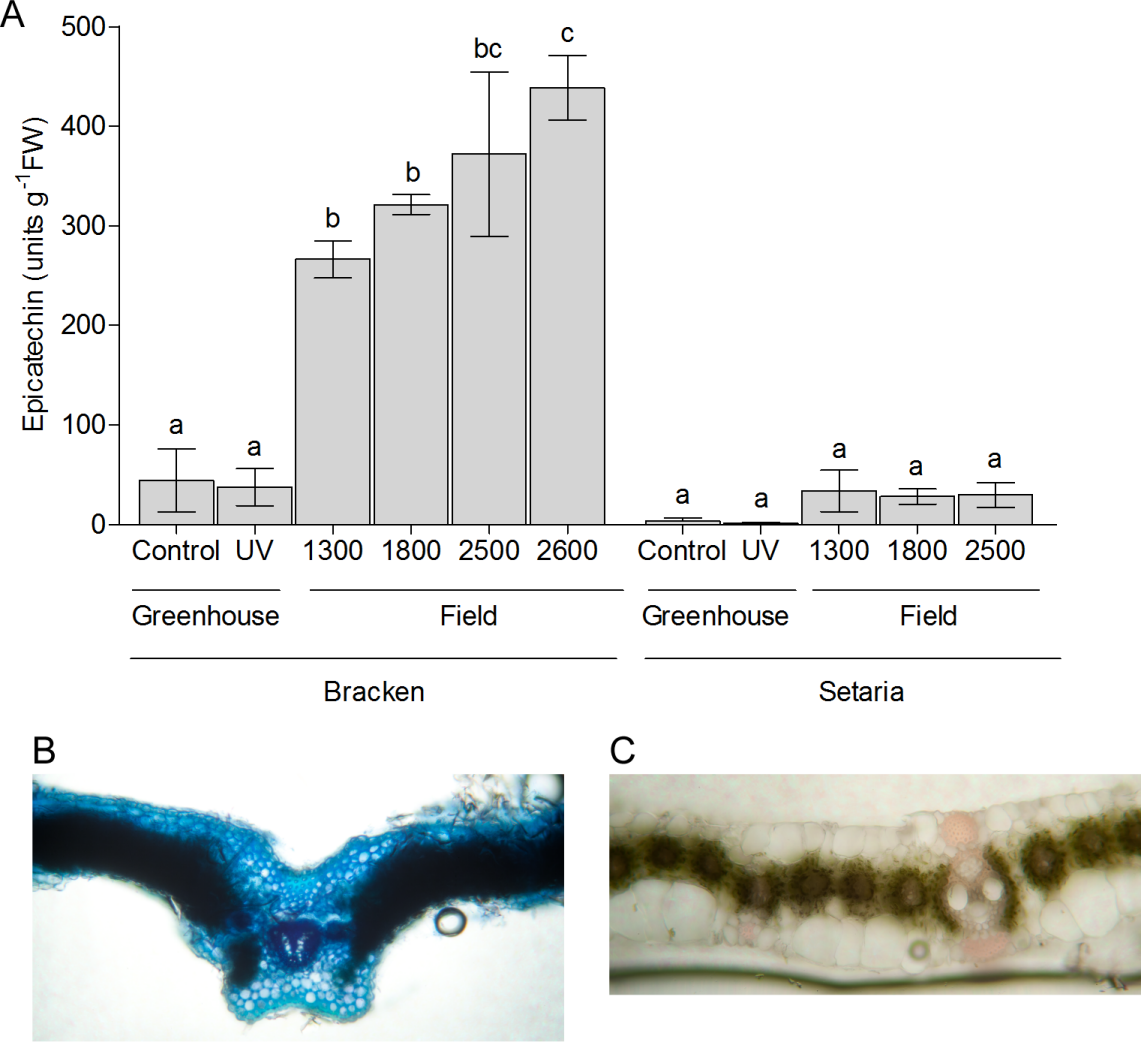
Proanthocyanidin contents in Bracken and Setaria grown in the pastures. **(A)** Condensed tannins were solubilized by acid butanolysis as described by Pang et al. [44]. Epicatechin was used as a standard to calculate the monomeric units of the proanthocyanidins. “Control” and “UV” are samples from the greenhouse. Numbers are indicating the elevation of field-grown plants in m a.s.l. The graph shows the mean ± SEM of at least three independent biological replicates. The letters a, b and c indicate the level of significant differences (p ≤ 0.05) towards each other. Significance was calculated by ANOVA followed by Tukey’s pairwise comparisons. Cross-sections of a Bracken frond **(B)** and a Setaria leaf **(C)** were stained with a solution of 1% DMACA and inspected under a light microscope after washing out surplus staining solution with water. Blue color indicates insoluble proanthocyanidin accumulation. A representative example of n≥3 experiments is shown.

Interestingly, the debris of the leaves after extraction of the soluble compounds, remaining as the insoluble material, was dark brown only in samples from field-grown Bracken. In contrast, Setaria yielded colorless debris (S4 Fig). Mass-spectrometric analysis of soluble extracts had already suggested the presence of proanthocyanidins. TLC with subsequent DMACA staining of Bracken extracts revealed indeed some oligomeric compounds, while the bulk was polymeric. In Setaria, none of these compounds were observed (S4 Fig). The insoluble compounds of field-grown Bracken were quantified after hydrolysis with HCl-butanol [52]. The solubilized material of Bracken produced a green color with ferric ions indicating that epicatechin-like monomers had been released from the condensed tannins [52]. Remarkably, the content of these epicatechin units increased almost twofold along the elevational gradient from 1300 to 2600 m a.s.l.. In Setaria, no significant amounts of epicatechin units from condensed tannins were detected (Fig 8A). The major response of Bracken to the changes of the environment with the increasing elevation was thus the formation of insoluble proanthocyanidins, whose cellular localization was analyzed in leaf cross-sections prepared from field material and stained with DMACA (Fig 8B). The deep blue color that appeared in thin sections of Bracken fronds, but not of Setaria leaves, points to the accumulation of these polymers in the cell walls where it can serve as an effective UV screen.

## Discussion

This work, to the best of our knowledge, is the first attempt to portray the differential success of two plant species along an environmental gradient by a model that has been parameterized with measured ecological and physiological data. Ecologically, the competition between Bracken and the pasture grass Setaria is of special interest, as it reflects the equipoise of two plant species of different modes of photosynthesis. Both species are light-demanding, and their photosynthetic carbon net uptake is saturated at or even beyond 1000 μmol quanta m^-2^s^-1^ of PAR (S1 Fig). Therefore, establishment of dense vegetation cover by one of the two species additionally weakens growth of the competitor by shadowing. An example of such effect is the ousting of Bracken by planting Balsa trees (*Ochroma pyramidale*) [53] whose seedlings obviously are resistant to the allelopathic effect of Bracken frond leachate [20]. Whether such allelopathic effect is the reason why the amply produced Setaria seeds do not germinate in the pastures of our research area, as has been shown for herbs and C3-photosynthesis type grasses [19], needs further investigation. However, growth of transplanted sections of Setaria tussocks is obviously not inhibited in soils where Bracken had previously been growing.

None of the two species are components of the original vegetation, the tropical mountain forest [54, 55]. Setaria has been introduced from tropical Africa as a pasture grass, and Bracken invades the sites as a weed following land reclamation [4]. Both, Bracken and Setaria are therefore not *per se* adapted to the environmental conditions of the area. The grass is planted with a large population size whereas the fern enters the area via a massive import of spores together with a potential invasion of rhizomes from adjacent infected sites. Both plants react initially positively to the common management of burning the pastures for rejuvenation, but recurrent burning stimulates the regrowth of Bracken more than that of the grass [3, 56]. In spite of a frequent cloud cover in the eastern ranges of the Andes, increasing elevation is associated with a decrease in temperature [7] and an increase in UV radiation [57, 58].

### Lower temperatures at higher elevations limit photosynthesis and growth of Setaria

Responses of photosynthetic CO_2_ net uptake to light intensity and CO_2_ concentration show a clear decrease of photosynthesis with increasing elevation for Setaria, but only a low impact on Bracken (Fig 2). This observation is corroborated by photosynthetic measurements of both plant species grown and measured at 10, 20, and 30°C (Fig 4A), as well as by the modeled temperature optimum using the SoBraCo model for carbon acquisition (Fig 4B). The analyzed temperature dependence of photosynthesis revealed that temperatures below 15°C (at light intensities of 500 μmol m^-2^ s^-1^) or 22°C (at saturating light of 1200 μmol m^-2^ s^-1^) favor Bracken against Setaria. Forcing the model with highly time-resolved microclimate data representing monthly averages over 16 years indicates a slightly superior growth of Setaria to that of Bracken on the lower pasture, but results in the opposite effect higher up in the mountains (Fig 3). Interestingly, the model showed also some expected seasonal trends during the year, namely that in the less humid months, C4-photosynthesis of Setaria was more efficient at 1835 m a.s.l.. Even at mid elevation (2110 m a.s.l.), there was still some advantage for Setaria, but only in the driest month November. Considering the entire year, the model indicates higher biomass production for Setaria than for Bracken at the lowest experimental area at 1835 m a.s.l., comparable growth for Setaria and Bracken at mid-elevation (2110 m a.s.l.), and a clear advantage of Bracken at 2825 m a.s.l. (Fig 3). Although the overall tendency of the field observation is reflected by the model, the actual substantial occurrence of Bracken at the lowest site suggests the impact of further environmental factors on Setaria, which in their sum shift the equilibrium between the two species in favor of the fern.

### Setaria is more sensitive than Bracken to UV radiation

On clear days, total UV-irradiance increases with elevation. UV-A increases by up to 9%, UV-B up to 24% per 1000 m altitudinal difference [57, 59, 60]. Cabrol et al. [60] showed for the Bolivian Andes typical UV-A values around 6 W/m^2^ at noon, while UV-B at the same time lies in a range of 0.5–1 W/m^2^. During times of high cloud cover, as particularly occurring in our study area [30], spikes of UV-B of up to 10 W/m^2^ had been reached around 9:00 a.m. local time. This “overradiation” has been proven to also occur for global radiation in our study area, most likely through multiple reflections from clouds [33]. Exposure of Setaria plants to UV radiation resulted in reduced photosynthetic quantum yield concomitant with a decrease of the photosynthetic key protein D2 and the compelling inactivation of PSII (Fig 5) as was already shown in other studies [61, 62]. In contrast, Bracken did not show any significant change in the photosynthetic capacity under increased UV exposure. The values for maximum quantum yield did not significantly change in comparison with control samples, suggesting that Bracken is able to adapt and perform well even at higher elevations.

The combined results from field measurements and corresponding laboratory studies suggest that the prevailing average temperature and the UV radiation on the pastures limit the growth of Setaria more severely than that of Bracken. In general, C4-plants exhibit a higher temperature optimum of CO_2_ fixation. Therefore, from the type of photosynthetic carbon capture, Setaria is less suited to successfully compete with Bracken at elevations with average temperatures below 20°C. At 1835 m a.s.l., the average annual temperature of 16°C is by 4°C lower than the reported optimal temperature for growth of Setaria [63] which is particularly adapted to sunny, hot and low-humidity regions. The combination of relatively low temperatures (14°C day/10°C night) and high irradiation in the tropical mountains favors photoinhibition of the C4-plant more than of a C3-plant [64] that is better adapted to moderate temperatures.

Both, Setaria and Bracken are equipped with a specific pattern of phenolic compounds (Fig 6, S2 and S3 Tables). In Bracken, their composition varies considerably with growth conditions and elevation of the locality, while in Setaria, the pattern of phenolic secondary compounds remains largely unchanged. The insensitivity of Bracken against UV irradiation resulting from its effective chemical UV screen can be considered as one of the reasons for its success in the tropical mountain pastures. Alonso-Amelot et al. [65] showed an increase in the content of condensed tannins with increasing elevation, but they did not demonstrate an ecophysiological effect of this phenomenon. While confirming their observation, we conclude that the insensitivity of Bracken against UV irradiation results at least partly from its effective chemical UV screen (Figs 6–8, S2 Table). Stress, in particular UV, is known to induce the formation of the phenylpropane protective system [66–69], especially in leaves [70], and our results suggest that Bracken is more effective in this respect (S3 Table). In contrast, Setaria did not show a comparable response to UV exposure what could be due to an already effective UV screen (S2 Table). However, the decrease of the photosynthetic capacity, together with the lower content of the D2 protein, proves that Setaria is not capable of producing a sufficiently effective UV screen and thus suffers from UV irradiation as well as potential ROS emergence when growing at higher elevations and suboptimal temperature (Figs 6–8; S2 and S3 Tables).

The protective efficacy and potential interactions of the bouquet of flavonoids and tannins in Bracken and Setaria are difficult to disentangle, but some general effects of major individual components may be highlighted in the following. Flavonoids as well as catechins as monomers of the condensed tannins are located as soluble compounds in the vacuoles, but upon polymerization become immobilized in the cell walls (blue color in Fig 8B). They absorb UV-A, UV-B and blue light (S3 Fig) and thus can effectively screen the mesophyll from energy-rich radiation. The screening effectiveness depends on the respective absorption coefficients and concentrations. Even in the untreated leaves, the absorbance of UV and shortwave blue light by soluble compounds is substantially higher in Bracken than in Setaria (S3 Fig). Flavonoids are also known as antioxidants or ROS scavengers in general and especially so, if they possess an ortho-dihydroxy substitution at the B-ring (hydroxyl groups at C3’ and C4’) like the flavonol quercetin, the flavon luteolin, the flavanols catechin and epicatechin and the O-glycosides of these compounds. Quercetin is the most effective antioxidant under the identified flavonoids as it provides two more reactive structural elements which can react with all kinds of radicals [71]. Together with its glycoside rutin it was found exclusively in Bracken grown under additional UV in the greenhouse and in the field, but not at all in Setaria. Fairly good but less effective antioxidants than the quercetin family compounds are the flavones luteolin and its derivative orientin which were found in Setaria independently from environmental impact, and the same holds for the flavonol kaempferol and its glycosides. The flavonoid bouquet of Bracken, too, showed antioxidants whose occurrence did not respond to the environmental conditions, namely various glycosides of kaempferol and the luteolin derivative orientin (S2 and S3 Tables). Apparently dependent on growth in the field is the occurrence of the anthocyanidin derivative. Cyanidin derivatives are known as very effective UV screens and antioxidants, however, their formation is also known as a symptom of phosphorus deficiency, especially in grasses. This interpretation is not unlikely, since the soils in the research area are poor in phosphate [28].

In addition to the soluble UV screens and antioxidants, Bracken leaves are protected by the condensed tannins in the cell walls. The flavanones hesperidin and poncirin as well as condensed tannins were found in field-grown Bracken, but not in the greenhouse-grown Bracken with additional UV treatment. This might be due to the fact that not UV alone, but in combination with lower temperatures or other environmental factors, is responsible for induction of these flavonoids. In contrast, the formation of protective compounds in Setaria seems to be generally limited, and an effective increase in the UV screen with the elevation could not be observed (Fig 6A, S2 Table). In summary, adaptive and non-adaptive chemical UV screens and antioxidants affect the fragile ecological equilibrium between Bracken and Setaria along the investigated elevational gradient.

## Outlook

One of the incentives for this study was the widespread loss of agricultural areas by the invasion of Bracken everywhere in the humid tropics. In the region of this study in the Andes of Southern Ecuador (Fig 1), the area of Bracken-infested pastures increased from 9% in 1975 to 34% in 2001, and of Bracken-infested, already abandoned land from 5% in 1985 to 9.5% in 2001 [72, 73]. Given the general competitive strength of tropical Bracken, the careful selection of useful plant species for cultivation in pastures is crucial. In neotropical pasture farming, grass species of the photosynthetic C4-type, like *Setaria sphacelata*, are used, e.g. *Pennisetum clandestinum* from Kenya in the lowlands (Kikuyu grass) [74], and *Melinis minutiflora* (molasses grass), an invasive species from tropical Africa [74, 75], or the indigenous but low yielding *Axonopus compressus* (blanket grass) [76]. However, with respect to biomass production, all of these species are inferior to Setaria, leading to a steady increase of the area of this pasture type. Our study shows that at lower elevations, a photosynthetic C4-type grass species like Setaria can successfully compete with Bracken, but in areas above 1800 m a.s.l., sustainable pasture farming requires grass species with biomass production and competitive strength that are less dependent on the environment, in particular on the ambient temperature. Therefore, the use of a C3-grass which like Bracken can thrive well also at lower temperatures is obvious. Scattered populations of the globally widespread and climatically as well as edaphically robust C3-grass species, *Holcus lanatus* (common velvet grass or Yorkshire Fog) [77] are found in the upland pastures of the research area [4]. Common velvet grass is native to Europe and Asia [78] but used globally in pasture farming due to its constancy of biomass production and high grazing tolerance. Propagating by seeds as well as by tillers, it can form grass swards up to a meter tall. In contrast to its published low forage value [79], palatability as well as crude fat, protein and metabolic energy content, the samples from the research area might well be more favorable than those of Setaria. Therefore, *Holcus lanatus* might be a candidate for pasture farming in Bracken-infested tropical uplands [80, 81]. Undoubtedly, achieving sustainability in tropical pasture farming will mitigate the pressure on the remaining natural mountain forest.

## Supporting information

S1 Fig Photosynthetic characterization of Bracken and Setaria in the field. (A) Light-response curves of photosynthetic CO_2_ net uptake (*A*) of Setaria (solid lines) and Bracken (dashed lines). *A* was determined as dependent on the light intensity (PAR). The light-saturation curves were taken at two different elevations: 1835 m (gray lines) and 2110 m (black lines). The measurements were performed at 400 μmol CO_2_ mol^-1^ and 25°C. (B) Photosynthesis rates were determined as dependent on CO_2_ partial pressure (C_i_). A/C_i_ curves were taken at two different elevations: 1835 m (gray lines) and 2110 m (black lines). The measurements were performed with 1200 μmol quanta m^-2^ s^-1^ and at 25°C (n ≥3). (modified from Knuesting et al., 2015)
(PDF)

S2 Fig Influence of supplemental UV in the greenhouse on growth and photosynthesis of Setaria and Bracken. Setaria (left) and Bracken (right) were exposed to UV light, with twelve 30-min periods of UV per day for 70 days (UV), and without additional UV-light exposure (Control). The plants were grown at 20°C.
(PDF)

S3 Fig Absorption spectra of methanolic leaf extracts. Aqueous HCl-MeOH extracts of greenhouse-grown Bracken (black lines) and Setaria (gray lines) without (broken lines) and with supplementary UV radiation (solid lines) were prepared and monitored with a UV/VIS spectrophotometer. Leaves of 3 experimental plants were ground in liquid nitrogen and extracted with 1 ml of MeOH:conc. HCl:H_2_O (90:1:1) (extraction medium per 0.1 g fresh weight. The samples were vortexed three times for 10 s in low light. The samples were then centrifuged for 5 min at 800 *xg*. The supernatants were collected and diluted (1:100) with the same solvent.
(PDF)

S4 Fig Proanthocyanidin contents in field-grown plants. (A) Colored pellets after extraction of soluble flavonoids. Origin of the samples is indicated with the corresponding elevations in m a.s.l.. (B) Separation of an aqueous HCl-MeOH extract by TLC (10 μl of the extract was loaded on a silica plate) and spraying the plate with 0.1% DMACA. Numbers are indicating the elevation of field-grown plants in m a.s.l..
(PDF)

S1 Table Geographical coordinates of the field sites. (PDF)

S2 Table MS-identified flavonoids of Setaria grown under greenhouse and field conditions. Flavonoids were extracted with aqueous HCl-MeOH and defatted with petrolether (E) or harvested with nanoparticles (N).
(PDF)

S3 Table MS-identified flavonoids of Bracken grown in greenhouse and under field conditions. Flavonoids were extracted with aqueous HCl-MeOH and defatted with petrolether (E) or harvested with nanoparticles (N).
(PDF)
