# Supplementary material for: Who will win where and why? An ecophysiological dissection of the competition between a tropical pasture grass and the invasive weed Bracken over an elevation range of 1000 m in the tropical Andes
Source: PLoS One. 2018 Aug 13;13(8):e0202255. doi: 10.1371/journal.pone.0202255 (PMC6089443; doi:10.1371/journal.pone.0202255)
Supplement: S1 Fig — (A) Light-response curves of photosynthetic CO2 net uptake (A) of Setaria (solid lines) and Bracken (dashed lines). A was determined as dependent on the light intensity (PAR). The light-saturation curves were taken at two different elevations: 1835 m (gray lines) and 2110 m (black lines). The measurements were performed at 400 μmol CO2 mol-1 and 25°C. (B) Photosynthesis rates were determined as dependent on CO2 partial pressure (Ci). A/Ci curves were taken at two different elevations: 1835 m (gray lines) and 2110 m (black lines). The measurements were performed with 1200 μmol quanta m-2 s-1 and at 25°C (n ≥3). (modified from Knuesting et al., 2015) (PDF) [file pone.0202255.s001.pdf]

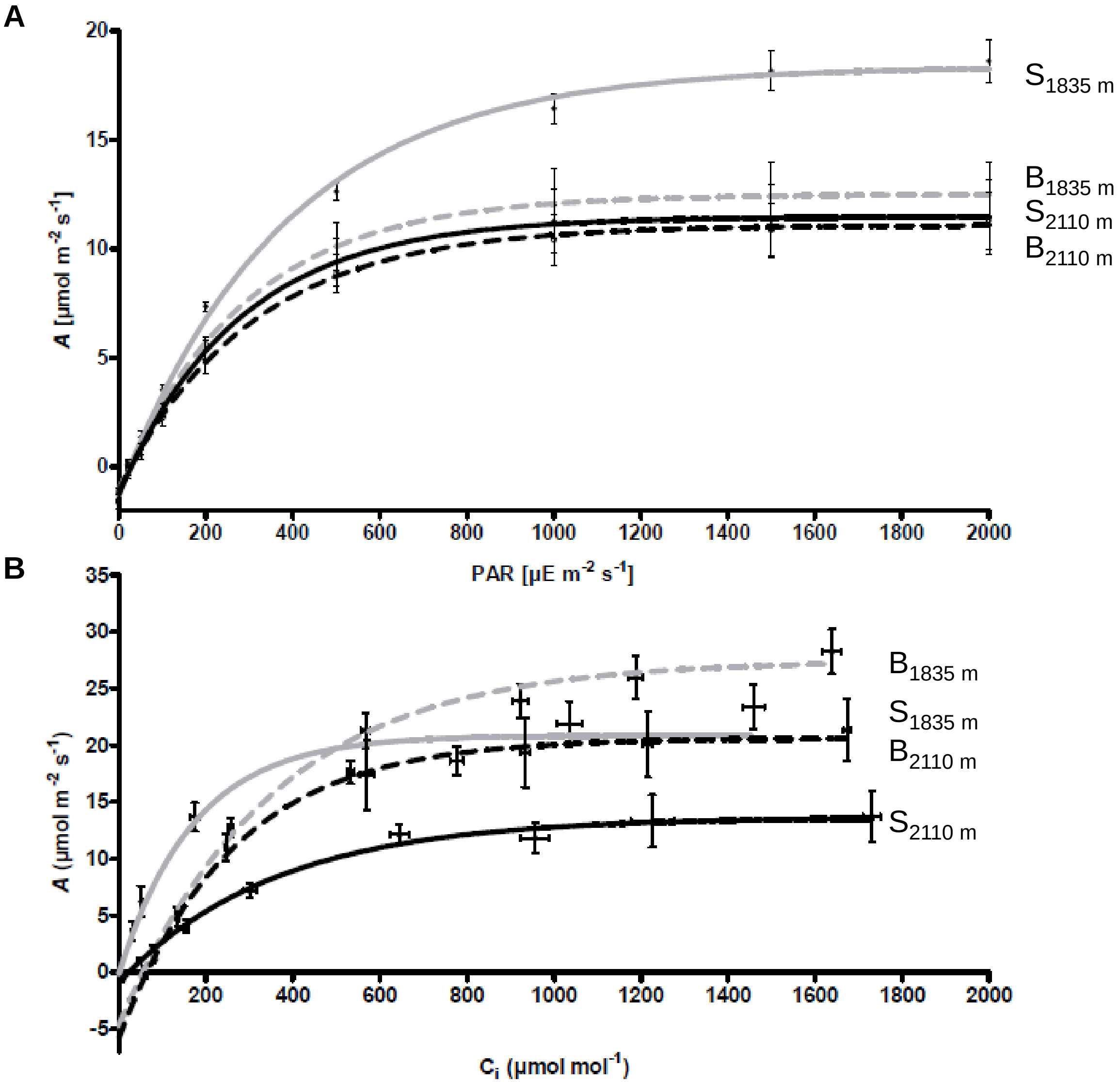

S1 Figure. Photosynthetic characterization of Bracken and Setaria in the field. (A) Light-response curves of photosynthetic CO_2 net uptake (*A*) of Setaria (solid lines) and Bracken (dashed lines). *A* was determined as dependent on the light intensity (PAR). The light-saturation curves were taken at two different elevations: 1835 m (gray lines) and 2110 m (black lines). The measurements were performed at 400 µmol CO_2 mol^{-1} and 25°C. **(B)** Photosynthesis rates were determined as dependent on CO_2 partial pressure (C_i). A/C_i curves were taken at two different elevations: 1835 m (gray lines) and 2110 m (black lines). The measurements were performed with 1200 µmol quanta m^{-2} s^{-1} and at 25°C (n ≥3). (modified from Knuesting et al., 2015)

Knuesting J, Schorsch M, Voss I, Brenner S, Bendix J, Strobl S, Beck E, Scheibe R (2015) Modeling the competition between the pasture grass *Setaria sphacelata* and Bracken: Comparison of photosynthetic capacities at different altitudes. *Tabebuia Bulletin* **1**(4): 16-19.
