# Supplementary material for: Who will win where and why? An ecophysiological dissection of the competition between a tropical pasture grass and the invasive weed Bracken over an elevation range of 1000 m in the tropical Andes
Source: PLoS One. 2018 Aug 13;13(8):e0202255. doi: 10.1371/journal.pone.0202255 (PMC6089443; doi:10.1371/journal.pone.0202255)
Supplement: S2 Fig — Setaria (left) and Bracken (right) were exposed to UV light, with twelve 30-min periods of UV per day for 70 days (UV), and without additional UV-light exposure (Control). The plants were grown at 20°C. (PDF) [file pone.0202255.s002.pdf]

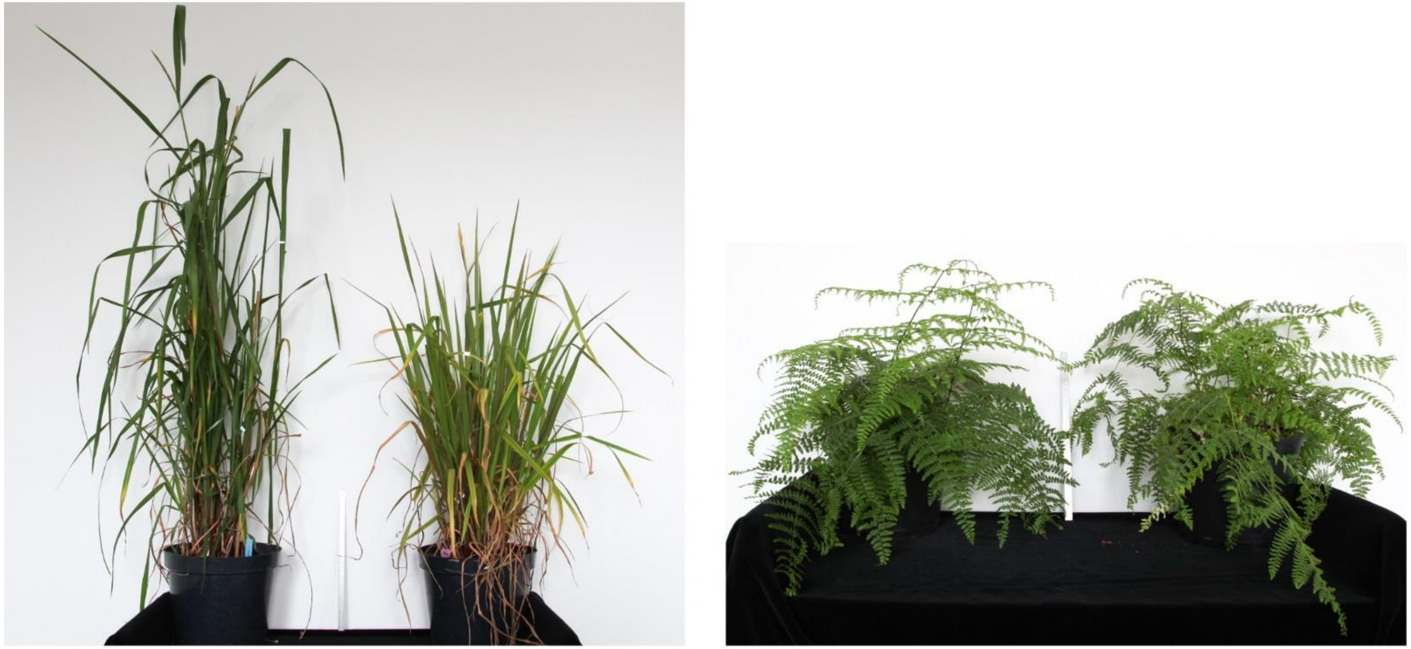

Control UV Control UV

S2 Figure. Influence of supplemental UV in the greenhouse on growth and photosynthesis of Setaria and Bracken. Setaria (left) and Bracken (right) were exposed to UV light, with twelve 30-min periods of UV per day for 70 days (UV), and without additional UV-light exposure (Control). The plants were grown at 20°C.
