# Supplementary material for: Who will win where and why? An ecophysiological dissection of the competition between a tropical pasture grass and the invasive weed Bracken over an elevation range of 1000 m in the tropical Andes
Source: PLoS One. 2018 Aug 13;13(8):e0202255. doi: 10.1371/journal.pone.0202255 (PMC6089443; doi:10.1371/journal.pone.0202255)
Supplement: S3 Fig — Aqueous HCl-MeOH extracts of greenhouse-grown Bracken (black lines) and Setaria (gray lines) without (broken lines) and with supplementary UV radiation (solid lines) were prepared and monitored with a UV/VIS spectrophotometer. Leaves of 3 experimental plants were ground in liquid nitrogen and extracted with 1 ml of MeOH:conc. HCl:H2O (90:1:1) (extraction medium per 0.1 g fresh weight. The samples were vortexed three times for 10 s in low light. The samples were then centrifuged for 5 min at 800 xg. The supernatants were collected and diluted (1:100) with the same solvent. (PDF) [file pone.0202255.s003.pdf]

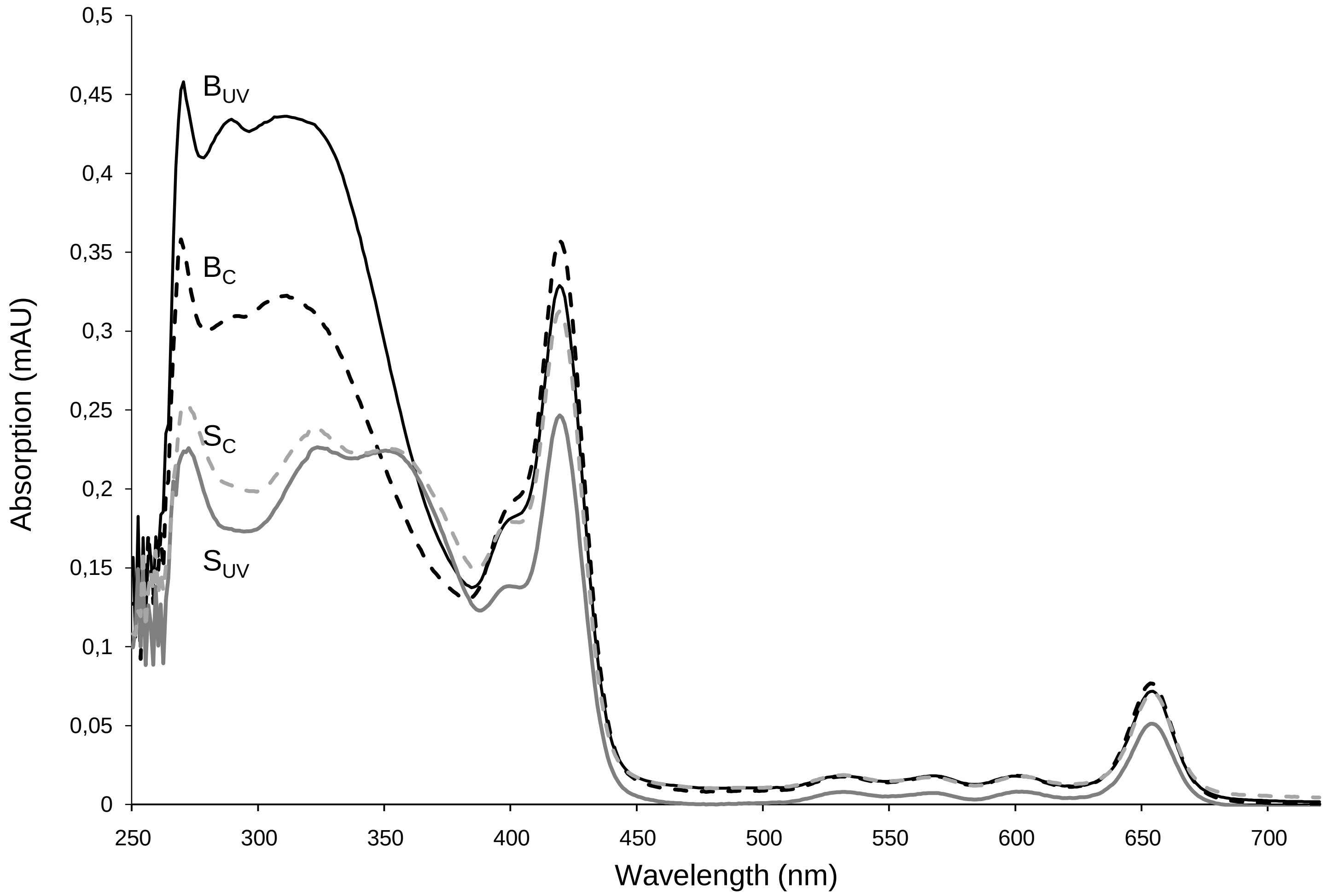

S3 Figure. Absorption spectra of methanolic leaf extracts. Aqueous HCl-MeOH extracts of greenhouse-grown Bracken (black lines) and Setaria (gray lines) without (broken lines) and with supplementary UV radiation (solid lines) were prepared and monitored with a UV/VIS spectrophotometer. Leaves of 3 experimental plants were ground in liquid nitrogen and extracted with 1 ml of MeOH:conc. HCl:H_2O (90:1:1) (extraction medium per 0.1 g fresh weight . The samples were vortexed three times for 10 s in low light. The samples were then centrifuged for 5 min at 800 *xg*. The supernatants were collected and diluted (1:100) with the same solvent.
