# Supplementary material for: Who will win where and why? An ecophysiological dissection of the competition between a tropical pasture grass and the invasive weed Bracken over an elevation range of 1000 m in the tropical Andes
Source: PLoS One. 2018 Aug 13;13(8):e0202255. doi: 10.1371/journal.pone.0202255 (PMC6089443; doi:10.1371/journal.pone.0202255)
Supplement: S4 Fig — (A) Colored pellets after extraction of soluble flavonoids. Origin of the samples is indicated with the corresponding elevations in m a.s.l.. (B) Separation of an aqueous HCl-MeOH extract by TLC (10 μl of the extract was loaded on a silica plate) and spraying the plate with 0.1% DMACA. Numbers are indicating the elevation of field-grown plants in m a.s.l.. (PDF) [file pone.0202255.s004.pdf]

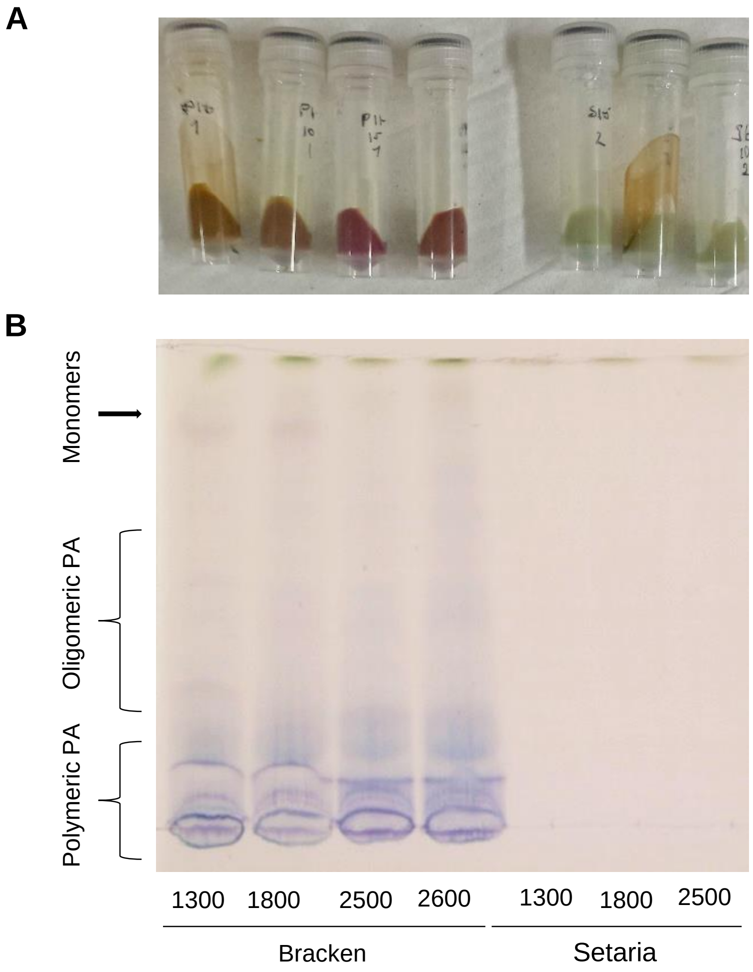


**S4 Figure. Proanthocyanidin contents in field-grown plants. (A)** Colored pellets after extraction of soluble flavonoids. Origin of the samples is indicated with the corresponding elevations in m a.s.l.. **(B)** Separation of an aqueous HCl-MeOH extract by TLC (10 µl of the extract was loaded on a silica plate) and spraying the plate with 0.1% DMACA. Numbers are indicating the elevation of field-grown plants in m a.s.l..
