# Supplementary material for: Who will win where and why? An ecophysiological dissection of the competition between a tropical pasture grass and the invasive weed Bracken over an elevation range of 1000 m in the tropical Andes
Source: PLoS One. 2018 Aug 13;13(8):e0202255. doi: 10.1371/journal.pone.0202255 (PMC6089443; doi:10.1371/journal.pone.0202255)
Supplement: S1 Table — (PDF) [file pone.0202255.s005.pdf]

S1 Table. Geographical coordinates of the field sites.

Site	Elevation (m a.s.l.)	Lon° (E)	Lat° (N)
ECSF	1835	-79.078730	-3.971359
Pasture	2110	-79.076820	-3.964252
El Tiro	2825	-79.144074	-3.986897
Site 1	1300	-79.007445	-4.028084
Site 2	1800	-79.078730	-3.972451
Site 3	2500	-79.127134	-3.983905
Site 4*	2600	-79.137015	-3.982028

*only Bracken
