# Supplementary material for: Who will win where and why? An ecophysiological dissection of the competition between a tropical pasture grass and the invasive weed Bracken over an elevation range of 1000 m in the tropical Andes
Source: PLoS One. 2018 Aug 13;13(8):e0202255. doi: 10.1371/journal.pone.0202255 (PMC6089443; doi:10.1371/journal.pone.0202255)
Supplement: S2 Table — Flavonoids were extracted with aqueous HCl-MeOH and defatted with petrolether (E) or harvested with nanoparticles (N). (PDF) [file pone.0202255.s006.pdf]

S2 Table. MS-identified flavonoids of Setaria grown under greenhouse and field conditions.

	Samples from greenhouse		Samples from field		
**Type of aglucon**	**Control**	**UV**	**1300 m**	**1800 m**	**2500 m**
Flavanon	Poncirin (N;E)	Poncirin (N;E)	Poncirin (E; N)	Poncirin (E; N)	Poncirin (E; N)
	---	Hesperidin (E)	---	Hesperidin (E; N)	Hesperidin (E; N)
Flavonol	Kaempferol-3-O-rutinoside (N)	Kaempferol-3-O-rutinoside (N)	Kaempferol-3-O-rutinoside (E)	K-3-O-rutinoside (E;N)	K-rut.(E;N)/K-7-O-neohesperid. (E)
Flavon	Luteolin (N)/Orientin (E)	Luteolin (N)/Orientin (E)	Luteolin (E)/Orientin (N)	Luteolin (N)/Orientin (E;N)	Luteolin (N)/Orientin (E)
	Tricin (N; E)	Tricin (N;E)	Tricin (E)	Tricin (E)	Tricin (E)
	Vitexin-2"-rhamnoside (N,E)	Vitexin-2"-rhamnoside (N;E)	Vitexin-2"-rhamnoside (E; N)	Vitexin-2"-rhamnoside (E)	Vitexin-2"-rhamnoside (E; N)/Vitexin (E; N)
	Saponarin (E)	Saponarin (E)	Saponarin (E)	---	Saponarin (E)
	Scoparin (E)	Scoparin (E)	---	---	---
Anthocyanidin conj	---	---	Cyanidin* (N)	Cyanidin* (N)	Cyanidin* (N)

Flavonoids were extracted with aqueous HCl-MeOH and defatted with petrolether (E) or harvested with nanoparticles (N).

* Cyanidin 3-O-[2''-O-(2'''-O-(sinapoyl) xylosyl) 6''-O-(p-O-(glucosyl) p-coumaroyl) glucoside] 5-O-glucoside, K = Kaempferol
