# Supplementary material for: Who will win where and why? An ecophysiological dissection of the competition between a tropical pasture grass and the invasive weed Bracken over an elevation range of 1000 m in the tropical Andes
Source: PLoS One. 2018 Aug 13;13(8):e0202255. doi: 10.1371/journal.pone.0202255 (PMC6089443; doi:10.1371/journal.pone.0202255)
Supplement: S3 Table — Flavonoids were extracted with aqueous HCl-MeOH and defatted with petrolether (E) or harvested with nanoparticles (N). (PDF) [file pone.0202255.s007.pdf]

S3 Table. MS-identified flavonoids of Bracken grown in greenhouse and under field conditions.

	**Samples from greenhouse**		**Samples from field**			
**Type of aglucon**	**Control**	**UV**	**1300 m**	**1800 m**	**2500 m**	**2600 m**
Flavanon	---	---	Hesperidin (E)	Hesperidin (E)	---	---
	---	---	---	---	Poncirin (E, N)	Poncirin (E)
Flavonol	K-3-O-rutinoside (N;E)	K-3-O-rutinoside (N;E)	K-7-O-neohesperidoside (N)	K-7-O-neohesperidoside (N)	K-7-O-neohesperidoside (N)	K-7-O-neohesp (N)
	---	---	---	---	K-3-O-rutinoside (E, N)	K-3-O-rutinoside (E,N)
	---	Quercetin(E)/ Rutin (E)	Quercetin (E)/Rutin (E,N)	Quercetin (E;N)/Rutin(E,N)	Quercetin (N)/Rutin(E,N)	Quercetin (N)/Rutin(E,N)
Flavon	Orientin (E)	Orientin (E)	Orientin (N)	Orientin (N)	Orientin (N)	---
Anthocyanidin conj	---	---	---	---	Cyanidin* (E)	Cyanidin* (E, N)
Condensed tannin	---	---	---	---	Procyanidin B1 (E)	---

Flavonoids were extracted with aqueous HCl-MeOH and defatted with petrolether (E) or harvested with nanoparticles (N).

* Cyanidin 3-O-[2''-O-(2'''-O-(sinapoyl) xylosyl) 6''-O-(p-O-(glucosyl) p-coumaroyl) glucoside] 5-O-glucoside, K = Kaempferol
